# The spectrum of covariance matrices of randomly connected recurrent neuronal networks with linear dynamics

**DOI:** 10.1371/journal.pcbi.1010327

**Published:** 2022-07-21

**Authors:** Yu Hu, Haim Sompolinsky

**Affiliations:** 1 Department of Mathematics and Division of Life Science, The Hong Kong University of Science and Technology, Hong Kong SAR, China; 2 Edmond and Lily Safra Center for Brain Sciences, The Hebrew University of Jerusalem, Jerusalem, Israel; 3 Center for Brain Science, Harvard University, Cambridge, Massachusetts, United States of America; Chinese Academy of Sciences, CHINA

## Abstract

A key question in theoretical neuroscience is the relation between the connectivity structure and the collective dynamics of a network of neurons. Here we study the connectivity-dynamics relation as reflected in the distribution of eigenvalues of the covariance matrix of the dynamic fluctuations of the neuronal activities, which is closely related to the network dynamics’ Principal Component Analysis (PCA) and the associated effective dimensionality. We consider the spontaneous fluctuations around a steady state in a randomly connected recurrent network of stochastic neurons. An exact analytical expression for the covariance eigenvalue distribution in the large-network limit can be obtained using results from random matrices. The distribution has a finitely supported smooth bulk spectrum and exhibits an approximate power-law tail for coupling matrices near the critical edge. We generalize the results to include second-order connectivity motifs and discuss extensions to excitatory-inhibitory networks. The theoretical results are compared with those from finite-size networks and the effects of temporal and spatial sampling are studied. Preliminary application to whole-brain imaging data is presented. Using simple connectivity models, our work provides theoretical predictions for the covariance spectrum, a fundamental property of recurrent neuronal dynamics, that can be compared with experimental data.

## Author summary

Here we study the distribution of eigenvalues, or spectrum, of the neuron-to-neuron covariance matrix in recurrently connected neuronal networks. The covariance spectrum is an important global feature of neuron population dynamics that requires simultaneous recordings of neurons. The spectrum is essential to the widely used Principal Component Analysis (PCA) and generalizes the dimensionality measure of population dynamics. We use a simple model to emulate the complex connections between neurons, where all pairs of neurons interact linearly at a strength specified randomly and independently. We derive a closed-form expression of the covariance spectrum, revealing an interesting long tail of large eigenvalues following a power law as the connection strength increases. To incorporate connectivity features important to biological neural circuits, we generalize the result to networks with an additional low-rank connectivity component that could come from learning and networks consisting of sparsely connected excitatory and inhibitory neurons. To facilitate comparing the theoretical results to experimental data, we derive the precise modifications needed to account for the effect of limited time samples and having unobserved neurons. Preliminary applications to large-scale calcium imaging data suggest our model can well capture the high dimensional population activity of neurons.

## 1 Introduction

Collective dynamics in networked systems are of great interest, with numerous applications in many fields, including neuroscience, spin glasses, social and ecological networks [1]. Many studies of neuronal networks have focused on how certain statistics of dynamics depend on the network’s connectivity structure [2–4], including the population average [5] and variance [6] of pairwise correlations. These dynamic features can be estimated experimentally by repeatedly recording a small number of neurons at each time. In this sense, they may be regarded as *local* or *marginal* features of dynamics. On the other hand, certain *global* or *joint* dynamic features may only be possible or efficient to estimate by recording a population of neurons *simultaneously*. An important example is the eigenvalues of the covariance matrix, which are complicated nonlinear functions of all the matrix elements. These eigenvalues arise naturally when performing the widely used Principal Component Analysis (PCA) of population activity, where they correspond to the amount of variance contained in each principal component of the activity. Although one can in principle fill out the covariance matrix through repeated pairwise recordings, the matrix is much more efficiently calculated from simultaneously recorded data. Furthermore, a sample covariance matrix calculated from simultaneously recorded data also requires particular methods to address the effect due to the finite recording length (Section 3.7) which would be different from repeated recordings. Another example of global dynamic features that has recently received substantial interest [7–11] is the effective dimensionality of neural population activity. When describing the data distribution in terms of linear subspaces, the dimensionality can be defined based on the moments of the covariance eigenvalues. Many recent experimental studies have observed a low dimensional dynamics of neurons in the brain [12, 13], and theoretical investigations have illustrated the importance of having a low dimensionality for brain function and computation [14], such as when representing stimuli [15] and generating motor outputs [13].

As the experimental techniques of measuring the activity of large population of neurons in biological networks become increasingly available, new opportunities arise for studying how the network’s connectivity structure affects these global aspects of population dynamics. In this work, we study the eigenvalue distribution (i.e., spectrum) of the covariance matrix of spontaneous activity in a large recurrent network of stochastic neurons with random connectivity. We focus on several basic and widely used models of random connectivity, including independent and identical Gaussian distributed connectivity [2] (Section 3.1), networks with second-order connectivity motifs [5, 16–20] (Section 3.3), and random Excitation-Inhibition (EI) networks (Section 3.5). Random connectivity has been a fundamental model in theoretical studies of neuronal network dynamics [2, 8, 21]. It can be motivated as a minimal model to capture the complex, disordered connections observed in many neuronal circuits, such as in the cortex. Some aspects of these covariance spectra might be distinct from those under ordered, deterministic connectivity (Section 4.2).

The dynamics considered here is simple where the activity fluctuations around the steady-state are described by a linear response [22, 23], which experimentally is related to spontaneous or persistent neural activity in absence of structured spatial-temporal stimuli. Despite the simple dynamics and minimal connectivity model, we find the resulting spectrum has a continuous bulk of nontrivial shape exhibiting interesting features such as a power-law long tail of large eigenvalues (Section 3.2), and strong effects due to the non-normality of the connectivity matrix (Section E.2 in S1 Text). These covariance spectra highlight interesting population-level structures of neuronal variability shaped by recurrent interactions that were previously unexplored.

Using the theory of the covariance spectrum, we derive closed-form expressions for the effective dimensionality (previously known for the simple random i.i.d. Gaussian connectivity [6]) We show that the continuous bulk spectrum has the advantage over low-order statistics such as the dimensionality thanks to its robustness to low rank perturbations (Section 3.3 to 3.5 and 3.8). Our analytically derived eigenvalue distributions can be readily compared to real activity data of recurrent neural circuits or simulations of more sophisticated computational models. We provide ready-to-use code to facilitate such applications (see **Data Availability Statement**). An example of such an application for a whole-brain calcium imaging data is presented in Section 3.8.

## 2 Model

### 2.1 Neuronal networks with random recurrent connectivity

We consider a recurrent network of linear rate neurons driven by noise

$$\begin{array}{c} \tau {\dot{x}}_{i}\left(t\right)=-x_{i}\left(t\right)+\sum_{j=1}^{N}J_{ij}x_{j}\left(t\right)+\xi_{i}\left(t\right),\;i=1,\ldots ,N. \end{array}$$ (1)

Here *x*_*i*_(*t*) is the firing rate of neuron *i*. *J*_*ij*_ describes the recurrent interaction from neuron *j* to *i*. *τ* is a time constant describing how quickly the firing rates changes in response to inputs. The network is driven by independent Gaussian white noise *ξ*_*i*_(*t*) with variance *σ*^2^, that is, the expectation 〈*ξ*_*i*_(*t*)*ξ*_*j*_(*t* + *τ*)〉 = *σ*^2^*δ*_*ij*_*δ*(*τ*).

We focus on the structure of long time scale covariation in the network, which are described by the *long time window* covariance $C_{ij}={\text{lim}}_{\Delta T\to \infty }\frac{1}{\Delta T}C_{ij,\Delta T}$. *C*_*ij*,Δ*T*_ is the covariance of the summed activity over a window of Δ*T*: *C*_*ij*,Δ*T*_ = 〈Δ*s*_*i*_(*t*)Δ*s*_*j*_(*t*)〉, $\Delta s_{i}\left(t\right)=\int_{t}^{t+\Delta T}\Delta x_{i}\left(t^{\prime }\right)dt^{\prime }$. For biophysical neurons, *C*_*ij*,Δ*T*_ typically settles to its limiting value when Δ*T* > 50ms [24]. It can be shown [25] that the long time window covariance *C* (also the zero-frequency covariance, see Section 3.6) is

$$\begin{array}{c} C=\sigma^{2}{\left(I-J\right)}^{-1}{\left(I-J\right)}^{-T}. \end{array}$$ (2)

Here *I* is the identity matrix, and *A*^−1^, *A*^*T*^ are the matrix inverse and transpose (*A*^−*T*^ = (*A*^−1^)^*T*^). For simplicity we will set *σ*^2^ = 1 unless stated otherwise. The covariance matrix *C* can also be estimated from experimental data consisting of simultaneously recorded neurons (Methods). We consider generalizations beyond the long time window covariance in Section 3.6.

Our analysis and results start from the covariance-connectivity relation Eq (2), which also describes, or closely approximates, the network dynamics in other models (Section 5.2) including networks of integrate-and-fire or inhomogeneous Poisson neurons [23, 26–28], fixed point activity averaged over whitened inputs, and structural equation modeling in statistics [29]. These models provide additional motivations for the covariance (Eq (2)), which may allow interpreting our results in experiments where the neural activity is driven by stimuli [10].

For many biological neural networks, such as cortical local circuits, the recurrent connectivity is complex and disordered. Random connectivity is a widely used minimal model to gain theoretical insights on the dynamics of neuronal networks [2, 4]. We first consider a random connectivity where

$$\begin{array}{c} J_{ij}∼N\left(0,g^{2}/N\right) \end{array}$$ (3)

are drawn as independent and identically distributed (i.i.d.) Gaussian variables with zero mean and variance *g*^2^/*N* (referred subsequently as the *i.i.d. Gaussian connectivity*). The covariance spectrum follows directly from results in random matrices [30, 31]. We then show how to generalize to other types of random connectivity, including: those with connectivity motifs (Section 3.3), Erdős-Rényi random connectivity, networks with excitation and inhibition (Section 3.5). The theory we derived assumes the network is large and is exact as *N* → ∞, and we verify their applicability to finite-size networks numerically. A list of variable notations is given in Table 1 for ease of reference.

**Table 1:** List of notations.

Notation	Description
*N*	number of neurons
*C*	covariance matrix, Eq (2)
*p*_*C*_(*x*)	pdf of eigenvalues of *C*, Section 2.2
*x*_±_	support edges of *p*_*C*_(*x*)
*J*	matrix of connection weights Section 2.1
*σ* ^2^	variance of white noise input, Section 2.1
*g*	connection strength var(*J*_*ij*_) = *g*^2^/*N*, (3)
*g*_*c*_	maximum *g* constrained by stability
*g*_*r*_	*g*/*g*_*c*_
*κ*	*ρ*(*J*_*ij*_, *J*_*ji*_) reciprocal motif cumulant, Eq (13)
*μ*	mean of eigenvalues $\frac{1}{N}\sum_{i=1}^{N}\lambda_{i}$
*D*	dimension, Eq (4)
*M*	number of time samples, Eq (37)
*α*	*N*/*M*, Section 3.7
*N*_*s*_	number of sampled neurons, Section 3.7
*f*	*N*_*s*_/*N*, Section 3.7

### 2.2 Covariance eigenvalues and dimensionality

Principal Component Analysis (PCA) is a widely used analysis of population dynamics, where the activity is decomposed along orthogonal patterns or Principal Components (PCs). The PCs are the eigenvectors of the covariance matrix *C* (Eq (37)), and the associated eigenvalues λ_*i*_ are nonnegative and show the amount of activity or variance distributed along the modes. In this work, we focus on the distribution of these covariance eigenvalues, described by the (empirical) probability density function (pdf) *p*_*C*_(*x*) which is defined through the equality $\int_{a}^{b}p_{C}\left(x\right)\left(x\right)dx=\frac{1}{N}\#\left\{\lambda_{i}\in \left(a,b\right]\right\}$ for all *a*, *b*. We also refer to *p*_*C*_(*x*) as the spectrum (which should not be confused with the frequency spectrum obtained via Fourier transform). We will derive the limit of *p*_*C*_(*x*) as *N* → ∞ and study how it depends on the connectivity parameters such as *g* = *N*var(*J*_*ij*_).

The shape of *p*_*C*_(*x*) can provide important theoretical insights on interpreting PCA. For example, it can be used to separate outlying eigenvalues corresponding to low dimensional externally driven signals from small eigenvalues corresponding to fluctuations amplified by recurrent connectivity interactions [32] (Section 3.8). the spectrum is also closely related to the effective *dimension* of the population activity. In many cases, the linear span of the activity fluctuations is full rank, *N*. Nevertheless, most of the variability is embedded in a much lower dimensional subspace. A useful measure of the effective dimension, known as the participation ratio [8, 33] is given by

$$\begin{array}{c} D\equiv \frac{{\left(\sum_{i=1}^{N}\lambda_{i}\right)}^{2}}{\sum_{i=1}^{N}\lambda_{i}^{2}}. \end{array}$$ (4)

which can be calculated from the first two moments of *p*_*C*_(*x*). We will also derive explicit expressions for *D* in random connectivity models.

## 3 Results

### 3.1 Continuous bulk spectrum with finite support

**Fig 1:**
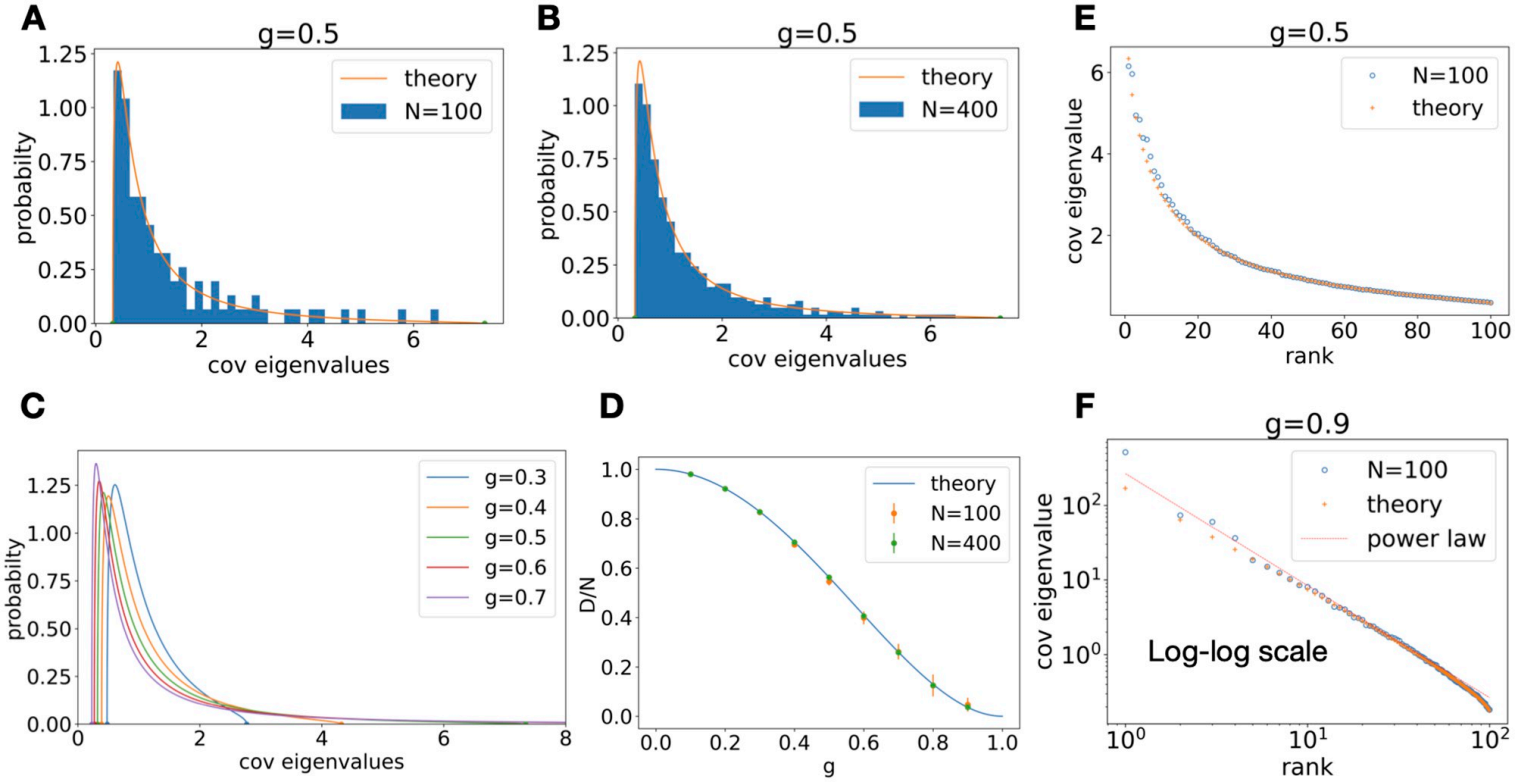
Covariance spectrum under random Gaussian connectivity. **A**. Compare theory (Eq (5)) with finite-size network covariance using Eq (2) at *N* = 100, *g* = 0.5. The histogram of eigenvalues is a single realization of the random connectivity. **B**. Same as A. at *N* = 400. **C**. Covariance eigenvalue distribution at various value of *g*. As *g* increases the distribution develops a long tail of large eigenvalues. **D**. Dimension (normalized by network size) vs *g*. The dots and error bars are mean and sd over repeated trials from finite-size networks (Eq (2) and use Eq (4)). Note some error bars are smaller than the dots **E**. Covariance eigenvalues vs. their rank (in descending order). The circles are covariance eigenvalues from a single realization of the random connectivity with *N* = 100 (Eq (2)). The crosses are predictions based on the theoretical pdf (Eq (5)). **F.** Same as E. but for *g* = 0.9 and on the log-log scale. The red dashed line is the power law with exponent −3/2 derived from Eq (5), see Section 3.2.

For networks with i.i.d. Gaussian connectivity (Section 2.1), there is one parameter *g* describing the overall connection strength. For stability of the fixed point and the validity of the linear response theory around it, *g* is required to be less than 1 [2]. The parameter *σ* in Eq (2) just scales all λ_*i*_ and thus is hereafter set to 1 for simplicity. Our main theoretical result is the following expression for the probability density function (pdf) of the covariance eigenvalues in the large *N* limit (Section A in S1 Text),

$$\begin{array}{cc} p_{C}\left(x\right)= & \;\frac{3^{\frac{1}{6}}}{2\pi g^{2}x^{2}}[\sum_{\xi =1,-1}\xi {\left(\left(1+\frac{g^{2}}{2}\right)x-\frac{1}{9}+\xi \sqrt{\frac{{\left(1-g^{2}\right)}^{3}x\left(x_{+}-x\right)\left(x-x_{-}\right)}{3}}\right)}^{\frac{1}{3}}],\\ & \;x_{-}\leq x\leq x_{+}. \end{array}$$ (5)

where

$$\begin{array}{c} x_{\pm }=\frac{2+5g^{2}-\frac{g^{4}}{4}\pm \frac{1}{4}g{\left(8+g^{2}\right)}^{\frac{3}{2}}}{2{\left(1-g^{2}\right)}^{3}}, \end{array}$$ (6)

and *p*_*C*_(*x*) = 0 for *x* > *x*_+_ and *x* < *x*_−_. The distribution has a smooth, unimodal shape and is skewed towards the left (Fig 1C). Near both support edges, the density scales as $|x-x_{\pm }{|}^{\frac{1}{2}}$ (Section A.1 in S1 Text).

The above result for the distribution *p*_*C*_(*x*) follows from the derivation of the circular law distribution of the eigenvalues of the random matrix *J* [30, 31, 34, 35]. However, to the best of our knowledge, this is the first exposition of the explicit expression for the spectrum of *C*, (Eq (5), which is essential for fitting to empirical data Section 3.8) and for the study of network dynamics. We emphasize that *p*_*C*_(*x*) does not have a *simple* relation to the spectrum of *J* because *J* is a non-normal matrix (i.e., *J*^*T*^*J* ≠ *JJ*^*T*^). This point is further elaborated in Section 3.3.2. Although the above result is derived in the large *N* limit, it matches accurately the spectrum of *C* in networks of sizes of several hundred, as demonstrated in our numerical results, Fig 1A and 1B (see also Fig A in S1 Text for additional realizations and trial-averages). In PCA and other analyses, the covariance eigenvalues are plotted in descending order vs. their rank [10, 36]. We can use the theoretical pdf Eq (5) to predict this *rank plot* by numerically solving the inverse cumulative distribution function (cdf), i.e., quantile function, at probability $\frac{N-\frac{1}{2}}{N},\frac{N-1-\frac{1}{2}}{N},\ldots ,\frac{\frac{1}{2}}{N}$. The closed form pdf (Eq (5)) allows for using the highly efficient Newton’s method to compute the quantiles. Fig 1E and 1F show a good agreement between the theory Eq (5) and a single realization of a *N* = 100 random network.

### 3.2 Long tail of large eigenvalues near the critical coupling

**Fig 2:**
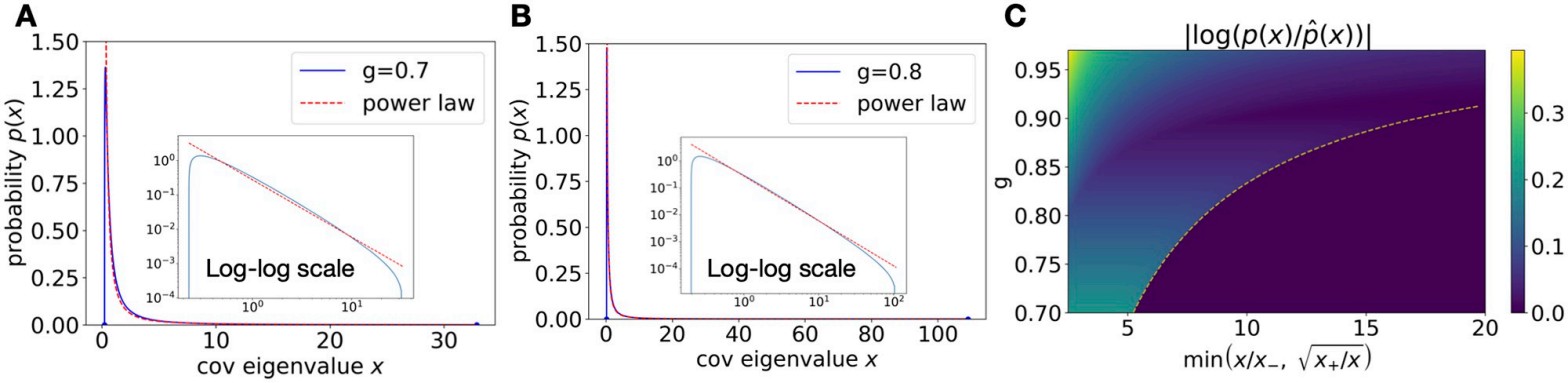
Approximate power-law tail. **A**. The exact pdf (solid line) of the covariance spectrum compared with the power-law approximation (dashed line, Eq (7)) at *g* = 0.7. Inset shows the log-log scale. **B**. Same as A. for *g* = 0.8. The approximation improves as *g* approaches the critical value 1. **C**. The log error between the exact pdf and approximation $|\text{log}\left(p\left(x\right)\right)-\text{log}\left(\hat{p}\left(x\right)\right)|$ as a function of *g* and “distance” from the support edges. We quantify this “distance” as the minimum ratio of *x*/*x*_−_ and $\sqrt{x_{+}/x}$ (more details and motivations in Section A.2 in S1 Text. The plot shows the log error is small when this ratio is large, which means *x* being far away from the edges. The dashed line shows the attainable region of the ratio which increases with *g*.

As *g* approaches the critical value of 1, the upper limit of the support *x*_+_ diverges as (1 − *g*^2^)^−3^ (Section 5.3 in Methods). This corresponds to an activity PC with diverging variance and is consistent with the stability requirement of *g* < 1. Note that the lower edge *x*_−_ is always bounded away from 0 and has a limit of $\frac{4}{27}$ as *g* → 1. Analyzing the shape of *p*_*C*_(*x*) for large *x* in the critical regime *g* → 1 yields a long tail of large eigenvalues, following a power law (Fig 2A and 2B, Methods)

$$\begin{array}{c} p_{C}\left(x\right)\approx \frac{\sqrt{3}}{2\pi }x^{-\frac{5}{3}}. \end{array}$$ (7)

As shown in Fig 2A, the power-law shape of *p*_*C*_(*x*) is apparent for *g* = 0.7 and does not require a *g* very close to 1.

To better elucidate the range of validity of the above power law, we consider the regime where 1 − *g*^2^ ≪ 1 and *x* ≫ 1. Define $z=\sqrt{1-x/x_{+}}$ where *x*_+_ ∝ (1 − *g*^2^)^−3^ is the upper edge of the support of *p*_*C*_(*x*). Then,

$$\begin{array}{c} p_{C}\left(x\right)\approx \frac{\sqrt{3}}{2\pi }x^{-\frac{5}{3}}2^{-\frac{1}{3}}F\left(z\right) \end{array}$$ (8)

where *F*(*z*) = (1 + *z*)^1/3^ − (1 − *z*)^1/3^. Thus, far from the spectrum upper edge, *z* → 1 and we obtain Eq (7), whereas near the upper edge *z* → 0 and $p_{C}\left(x\right)\approx \frac{3^{-\frac{1}{2}}2^{-\frac{1}{3}}}{\pi }x_{+}^{-\frac{5}{3}}\sqrt{1-x/x_{+}}$, which is the expected square-root singularity near the edge.

The power-law approximation of the probability density function Eq (5) translates to an approximation for the cumulative distribution function $F_{C}\left(x\right)\approx 1-\frac{3\sqrt{3}}{4\pi }x^{-\frac{2}{3}}$ This also means a power law in the rank plot has an exponent of −3/2 when connection strength *g* is close to the critical value (Fig 1F), providing an alternative mechanism based on recurrent circuits for the experimental observations of power-law distributed covariance eigenvalues in the literature [10, 36] (see also the model Eq 31 and discussions in Methods).

Because the probability density is small in the power-law tail, large eigenvalues can appear to be sparsely located (Fig 1A) and potentially mistaken for statistical outliers. This underscores the importance of knowing the exact distribution and support edges for interpreting PCA results of population activity, topics which we revisit later (Section 3.8). Note that a long tail in the spectrum is a distinct feature of correlations arising from the recurrent network dynamics (see also a heuristic explanation in Section E.3 in S1 Text). For example, for the Marchenko–Pastur law that is often used for modeling empirical covariance spectra, the upper edge of its support relative to the mean is bounded by 4 (Methods). In contrast, the same ratio for *p*_*C*_(*x*) (Eq (5)) can be arbitrarily large as *O*((1 − *g*^2^)^−2^) (see below for calculating the mean). This highlights the difference between covariance generated by finite samples of noise and correlations generated by the recurrent dynamics.

The long tail of the eigenvalue distribution is also reflected by a low effective dimension (Eq 4). In Section B in S1 Text we show that the mean and second moment of the eigenvalue distribution above, are given by

$$\begin{array}{c} E(\lambda )={\left(1-g^{2}\right)}^{-1},\;\;E(\lambda^{2})={\left(1-g^{2}\right)}^{-4}. \end{array}$$ (9)

which yields for the dimension

$$\begin{array}{c} D=N{\left(1-g^{2}\right)}^{2}. \end{array}$$ (10)

In particular, the *relative dimension* with respect to the network size *D*/*N* vanishes as *g* approaches 1 (Fig 1D). In comparison, *D*/*N* for the Marchenko–Pastur law (Eq 35) is at least $\frac{1}{2}$.

While these low-order moments can be derived from previous methods (see [6] and Section B in S1 Text, and also a parallel work [37]), our method allows for the derivation of higher-order moments, such as,

$$\begin{array}{c} E(\lambda^{3})={\left(1-g^{2}\right)}^{-7}\left(1+2g^{2}\right),\;E(\lambda^{4})={\left(1-g^{2}\right)}^{-10}\left(1+g^{2}\right)\left(1+5g^{2}\right). \end{array}$$ (11)

and in general,

$$\begin{array}{c} E(\lambda^{n})\propto {\left(1-g\right)}^{-3(n-1)-1},\;\;\text{as}\;g\to 1^{-} \end{array}$$ (12)

### 3.3 Impact of the asymmetry in connectivity

We next consider generalizations of random connectivity beyond the i.i.d. Gaussian model (Sction 2.1). An important feature of biological neural networks is the presence of motif structures at various scales [16–18, 38], which correspond to an overabundance of certain subgraphs, relative to their frequency in an edge-shuffled network (i.e., an i.i.d. random graph with matching connection probability). Using a random connectivity model with Gaussian distributed entries [38] Section C.1 in S1 Text, we can study the effects of the four types of second order motifs (i.e., consisting of two edges). Among them, the diverging, converging, and chain motifs correspond to a low-rank component *L* of the connectivity $J=L+\tilde{J}$, where the remaining part $\tilde{J}$ contains only reciprocal motifs. Based on the results in Section 3.4, we can focus below on studying the covariance spectrum under the simpler connectivity $\tilde{J}$ with only reciprocal motifs, because the bulk spectrum of $J=L+\tilde{J}$ is remains the same when adding the low-rank *L* (see examples in Section 3.4 and Section C in S1 Text). Note that the magnitude of diverging, converging, and chain motifs in the original connectivity still indirectly affect the bulk covariance spectrum by affecting the parameters *g* and *κ* (see below) of $\tilde{J}$ (see Section C.1 in S1 Text for the details of this relation).

For ease of notation, we still use *J* to represent a random connectivity matrix with potentially reciprocal motifs ($\tilde{J}$ described above). This is equivalent to varying the degree of asymmetry of *J* [31]. In this case, each component of *J* is $J_{ij}∼N\left(0,g^{2}/N\right)$ but *J*_*ij*_ and *J*_*ji*_ are correlated,

$$\begin{array}{c} \kappa =\rho (J_{ij},J_{ji}),\; \end{array}$$ (13)

with −1 ≤ *κ* ≤ 1. All other correlations are zero.

#### 3.3.1 Symmetric and anti-symmetric random networks

First, we consider two extreme cases for the reciprocal motifs: *κ* = 1 corresponding to *J*_*ij*_ = *J*_*ji*_, and *κ* = −1 corresponding to anti-symmetric matrix (or skew-symmetric *J*_*ij*_ = −*J*_*ji*_). These cases are much simpler to analyze, because *J* is a *normal matrix* so *p*_*C*_(*x*) can be derived from the well known eigenvalue distribution of *J* [31]. For symmetric random connectivity,

$$\begin{array}{c} p_{C,g,\kappa =1}\left(x\right)=\frac{\sqrt{\left(4g^{2}-1\right)x-1+2\sqrt{x}}}{4\pi g^{2}x^{2}},\;\;x\in \left(x_{-},x_{+}\right),\;x_{\pm }={\left(1\mp 2g\right)}^{-2}. \end{array}$$ (14)

Here stability requires that $g<\frac{1}{2}$. For anti-symmetric random connectivity,

$$\begin{array}{c} p_{C,g,\kappa =-1}\left(x\right)=\frac{\sqrt{(4g^{2}+1)x-1}}{2\pi g^{2}x^{2}\sqrt{1-x}},\;\;x\in \left({\left(1+4g^{2}\right)}^{-1},1\right). \end{array}$$ (15)

Here the network is stable for all *g*. The derivations are given in the Section D in S1 Text.

**Fig 3:**
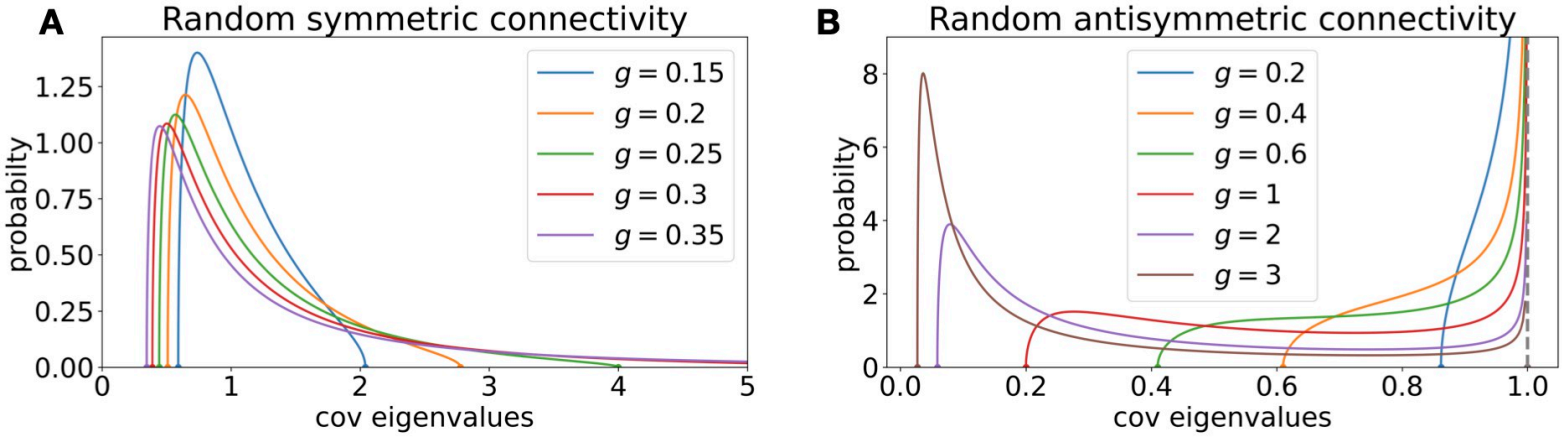
Covariance spectrum for the symmetric and anti-symmetric random connectivity. **A.** The pdf of a covariance spectrum with random symmetric *J* with different *g* (note $g<\frac{1}{2}$ for stability). **B.** Same as A., but for random anti-symmetric *J*_*ij*_ = −*J*_*ji*_. The pdf diverges at *x* = 1 as ${\left|1-x\right|}^{-\frac{1}{2}}$.

Since the general shape and trend depending on *g* of the spectrum in the simpler symmetric case (Fig 3A) is qualitatively similar to the i.i.d. case (Fig 1C), one can use it to gain intuition, for example, of the thin tail of large eigenvalues as *g* approaches its critical value.

Quantitatively using the equations above, we see that *p*_*C*_(*x*) of the symmetric random network (Fig 3A) has a power-law tail analogous to Eq (7) as *g* → 1/2 (i.e., large *x*) but with a different exponent from the i.i.d. case (Eq 7),

$$\begin{array}{c} p_{C,g,\kappa =1}\left(x\right)\approx \frac{\sqrt{2}}{\pi }x^{-\frac{7}{4}}. \end{array}$$ (16)

The *p*_*C*_(*x*) of the anti-symmetric random network (Fig 3B) does not have a long tail as the upper limit of the support is always 1. Since *J* here is a normal matrix, this qualitative difference from the i.i.d. random connectivity can be understood considering the eigenvalues of *J* [31]. The eigenvalues of *J* all lie on the imaginary axis and therefore never approach 1. Thus, the eigenvalues of the covariance matrix do not develop a long tail when increasing *g*.

#### 3.3.2 Connectivity with general asymmetry

**Fig 4:**
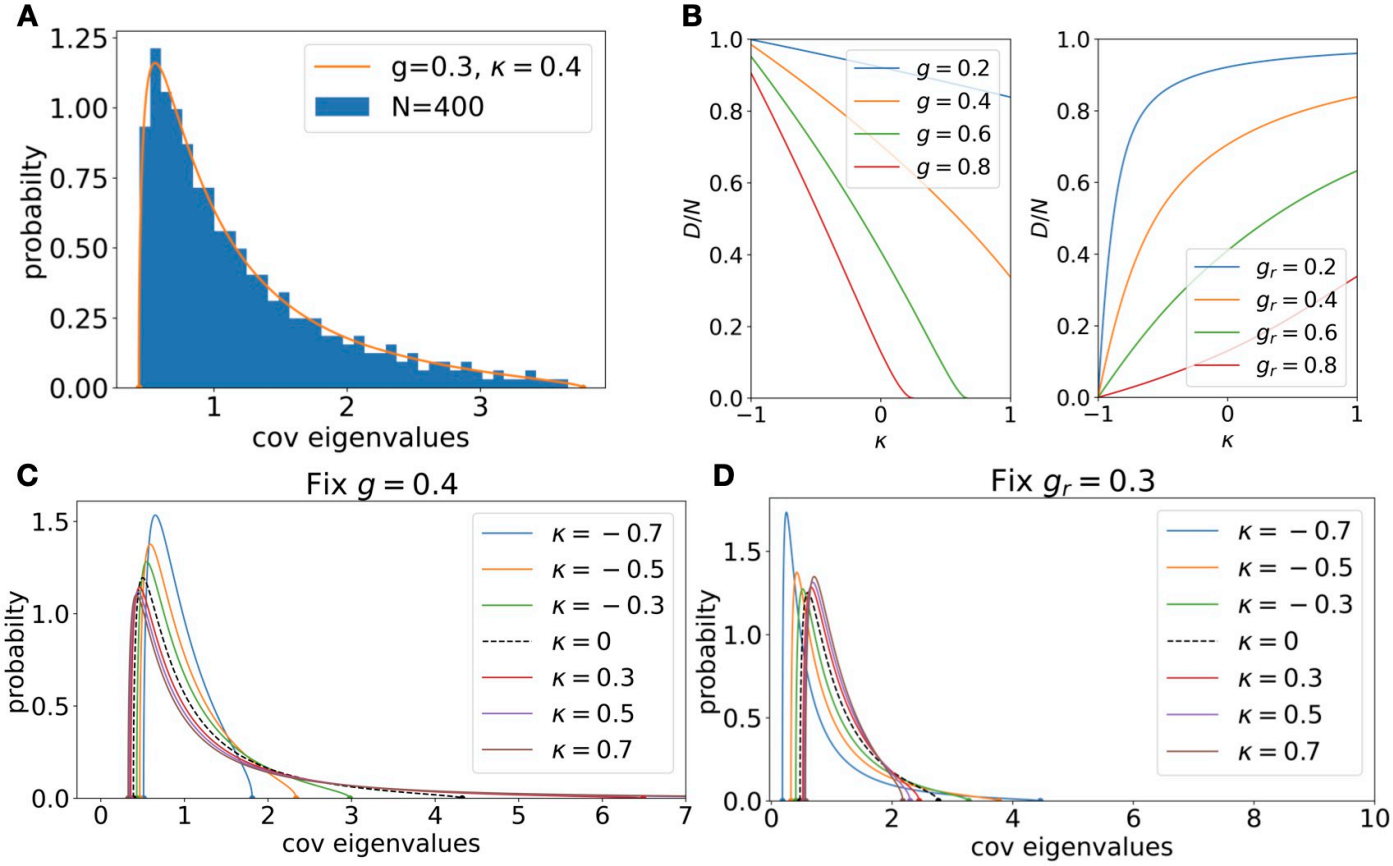
Impact of reciprocal motifs. **A**. Compare theoretical covariance spectrum for random connectivity with reciprocal motifs and a finite-size network covariance using Eq (2)(*g* = 0.4, *κ* = 0.4, *N* = 400). **B**. The impact of reciprocal motifs on dimension for various *g*_*r*_ = *g*/*g*_*c*_ (Eq (18)). For small *g*_*r*_, the dimension increases sharply with *κ*. **C**. The spectra at various *κ* while fixing *g* = 0.4. The black dashed line is the i.i.d. random connectivity (*κ* = 0). **D**. Same as C. but fixing relative *g*_*r*_ = 0.4 to control the main effect (see text). The changes in shape are now smaller and the support narrows with increasing *κ*.

For the Gaussian random connectivity with *κ* = *ρ*(*J*_*ij*_, *J*_*ji*_), −1 < *κ* < 1, we have derived an implicit equation for *p*_*C*,*g*,*κ*_(*x*) in the large *N* limit based on the results in [31] (Section E in S1 Text). Although a closed-form expression can be derived using the root formula for quartic equations, it seems quite cumbersome, hence we show here the numerical solutions of this equation. For a fixed *g*, as *κ* increases, the distribution broadens on both sides (Fig 4C). Intuitively, these effects (also Fig 4B) can be understood due to the change of the critical *g* for stability, which is now given by *g*_*c*_ = (1 + *κ*)^−1^ (based on the spectrum of *J* [31]). As *κ* increases, the *relative coupling strengthg*_*r*_ = *g*/*g*_*c*_ = *g*(1 + *κ*), which is also the maximum real part of *J*’s eigenvalues [31] increases, and the shape of the spectrum changes similar to increasing *g* in the case of i.i.d. connectivity (Fig 1C). This motivates us to further compare the distributions *p*_*C*,*g*,*κ*_(*x*) with the same *g*_*r*_ to study effects due to *κ* beyond changing *g*_*r*_. As shown in Fig 4D, when fixing the relative strength *g*_*r*_ the distribution narrows as *κ* increases.

Consistent with the above results, we have shown (Section E.1 in S1 Text) that for all intermediate values of −1 < *κ* < 1, the critical covariance spectrum has an asymptotic power-law tail with the same exponent as the i.i.d. random case (Eq (7))

$$\begin{array}{c} p_{C}\left(x\right)\approx \frac{\sqrt{3}}{2\pi }{\left(1-\kappa \right)}^{\frac{1}{3}}\left(1+\kappa \right)x^{-\frac{5}{3}},\;\text{as}\;x\to \infty ,\;g\to g_{c}={\left(1+\kappa \right)}^{-1} \end{array}$$ (17)

In comparison, the *κ* = ±1 are singular cases in Eq 17 and have a different limiting power-law behavior (an exponent of $-\frac{4}{7}$ and no long tail). This is intuitively consistent with the spectrum of *J* which becomes confined on a line for *κ* = ±1 rather than in an ellipses for −1 < *κ* < 1 [31] (see Section E.1 in S1 Text for more discussion).

The shape changes of *p*_*C*,*g*,*κ*_(*x*) with reciprocal motifs are also reflected by the dimension measure, for which we derived a closed-form expression (Section E in S1 Text)

$$\begin{array}{c} D=N\frac{\mu_{1}\sqrt{1+4(g^{2}-\theta )}}{{\left(\theta \mu_{1}+1\right)}^{2}\left(g^{2}\mu_{1}+1\right)},\;\;\mu_{1}=\frac{2\theta -1+\sqrt{1+4(g^{2}-\theta )}}{2(g^{2}-\theta^{2})},\;\theta =g^{2}\left(1+\kappa \right). \end{array}$$ (18)

Here *μ*_1_ is the mean of the distribution. Comparing with Eq 10, this shows the nontrivial dependence of dimension on the reciprocal motif strength *κ*. As *g* → *g*_*c*_ = (1 + *κ*)^−1^, $g^{2}-\theta^{2}=g^{2}\left(1-g_{r}^{2}\right)\to 0$. The numerator of *μ*_1_ is at least 2*θ* − 1 ≥ 2/(1 + *κ*) − 1 > 0. Therefore *μ*_1_ diverges as $O({\left(1-g_{r}^{2}\right)}^{-1})$. Since 1 + 4(*g*^2^ − *θ*) ≥ 1 + 4(*g*^2^ − *g*) ≥ (1 − 2/(1 + *κ*))^2^ > 0, we have *D*/*N* vanishes as $O\left(\mu_{1}^{-2}\right)=O\left({\left(1-g_{r}^{2}\right)}^{2}\right)$. The above limits under the critical *g* are similar to the *κ* = 0 case, Eq (10). Consistent with the shape changes, the dimension decreases with reciprocal motifs when fixing *g* and increases when fixing *g*_*r*_ (Fig 4B).

The general asymmetric random connectivity also provides an example of the strong effect of *J* being a non-normal matrix on the covariance spectrum. By continuity, one may expect that as *κ* decreases towards −1, the shape of *p*_*C*,*g*,*κ*_(*x*) will become similar to that of the anti-symmetric network *p*_*C*,*g*,*κ* = −1_(*x*), which is bimodal for sufficiently large *g* (i.e., has another peak in addition to the divergence at 1, Fig 3B). Indeed, assuming a normal *J* predicts a covariance spectrum that is bimodal with a non-smooth peak in a large region of −1 < *κ* < 0 and *g* (Fig H in S1 Text). Intriguingly, the actual spectrum *p*_*C*,*g*,*κ*_(*x*) is unimodal for all but a minuscule region of (*κ*, *g*) where *κ* < −0.95, indicating a suppression of a bimodal spectrum under negative *κ* due to the non-normal *J* (Section E.2 in S1 Text).

### 3.4 Adding low rank connectivity structure

**Fig 5:**
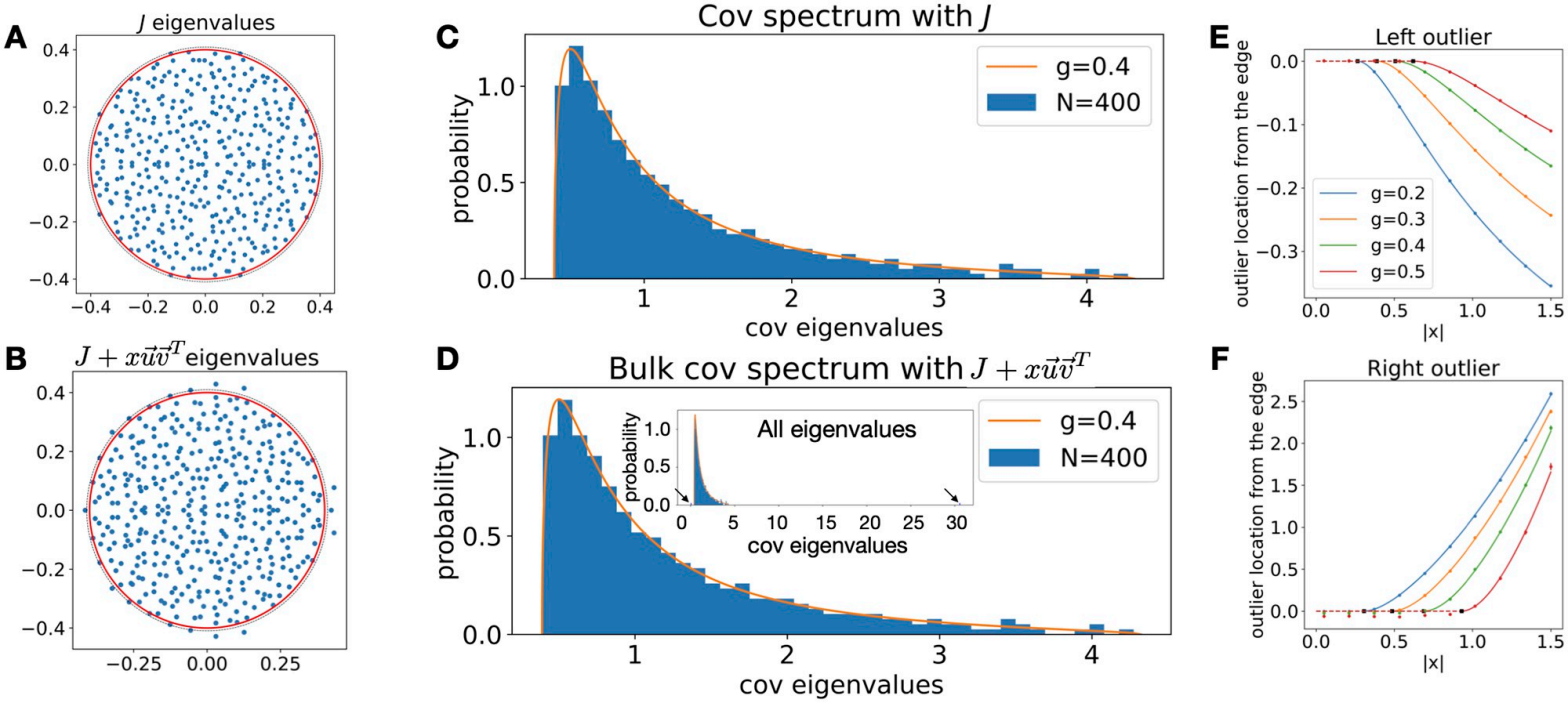
Robustness of the covariance spectrum to low rank perturbations of the connectivity. **A**. Eigenvalues of a Gaussian random connectivity *J* (Eq (3)), *g* = 0.4, *N* = 400. As *N* → ∞, the limiting distribution of eigenvalues is uniform in the circle with radius *g* ([34] red solid line). The black dashed line is the 0.995 quantile of the eigenvalue radius calculated from 1000 realizations. **B**. Same as A. but for the rank-1 perturbed *J* + *xuv*^*T*^. $u={\left(1,1,\ldots ,1\right)}^{T}/\sqrt{N}$, $v_{i}∼\;\text{i.i.d.}\;N\left(0,1/N\right)$ and *x* = 4.03. This example also corresponds to adding diverging motifs (Section 3.3 and Section C.1 in S1 Text). **C**. The histogram of covariance eigenvalues (Eq (2)) under the *J* in A. **D**. The bulk histogram of eigenvalues with *J* + *xuv*^*T*^ has little change and remains well described by the Gaussian connectivity theory (red line, Eq (5)). Besides the bulk, there are two outlier eigenvalues to the left and right (inset, arrows) **E,F**, Analytical predictions (solid and dashed lines) of the outlier locations given *g* and |*x*| when *u*, *v* are (asymptotically) orthogonal unit vectors that are independent of *J* (see other cases in Section C.3 in S1 Text). The y-axis is the outlier location subtracting the corresponding edge *x*_±_, Eq (6), so it is zero for small |*x*| before the outlier emerges (dashed line). The dots are the mean of the smallest (for the left outlier) or largest (right outlier) eigenvalues averaged across 100 realizations of the random *J*, *N* = 4000. The errorbars are the standard error of the mean (SEM, many are smaller than the dots).

An important property of the spectrum of *C* is the robustness of its bulk component to the addition of low rank structured connectivity. Many connectivity structures that are important to the dynamics and function of a recurrent neuronal network can be described by a full rank random component plus a low rank component [39, 40]. For example, such components may arise from Hebbian learning [41] and by training neural networks by gradient decent [42]. A simple case is where we add a rank *k* structured matrix that is deterministic or independent to the random component [43, 44]. As shown in Section C in S1 Text, in large networks, the bulk covariance spectrum remains unchanged, but the low rank component may give rise to at most 2*k* outlying eigenvalues. This is illustrated by the example of rank-1 perturbation to *J* with i.i.d. Gaussian entries in Fig 5C and 5D, where the expected location of the outliers in the covariance spectrum can be predicted analytically (Fig 5E and 5F and Section C.3 in S1 Text). This is in contrast to the spectrum of *J*, where the same perturbations can lead to an unbounded number of randomly located eigenvalues [43, 45] (Fig 5A and 5B). In sum, the bulk spectrum of covariance is robust against low rank perturbations to the connectivity. Note, however, the relevance of the bulk spectrum for the network dynamics depends on the location of outliers. Outliers to the right of the bulk spectrum may indicate potential instability of the dynamics even for *g* < 1, as discussed in the example below.

### 3.5 Sparse excitatory-inhibitory networks

The Gaussian random connectivity has a non-zero connection weight for all pairs of neurons with probability 1, where many biological networks are sparsely connected. In addition, each neuron has both excitatory (positive) and inhibitory (negative) weights, in contrast to many neuronal networks that obey Dale’s Law, namely all neurons are either excitatory (with all outgoing weights positive) or inhibitory (with negative outgoing weights). We consider here a simple model of E-I network, consisting of *N*/2 excitatory and *N*/2 inhibitory neurons. The probability of each connection *J*_*ij*_ to be nonzero, which may depend on the types of neurons *i* and *j*, is *K*_*αβ*_/*N*, *α*, *β* = *E*, *I*. Thus, the mean number of inputs to a neuron of type *α* from a population of type *β* is *K*_*αβ*_/2. All excitatory non-zero connections are of strength $w_{0}/\sqrt{K_{\alpha \beta }}$ and the inhibitory connections are $-w_{0}/\sqrt{K_{\alpha \beta }}$. We assume that *K*_*αβ*_ = *k*_*αβ*_*K* where *k*_*αβ*_ = *O*(1) and *K* ≪ *N*.

**Fig 6:**
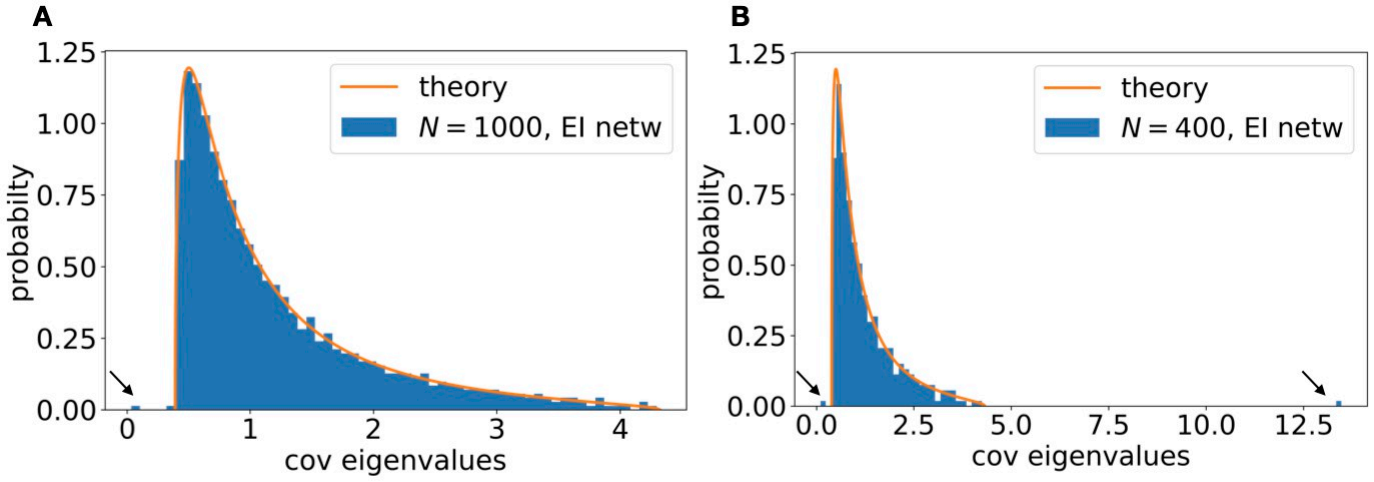
EI networks. **A**. One realization of the covariance eigenvalues by Eq (2) with an EI network satisfying the stability condition (see text). The bulk spectrum is well described by the Gaussian random connectivity theory (solid line, Eq (5)). There is one small outlier to the left of the bulk (arrow). The parameters are *g* = *w*_0_ = 0.4, $\sqrt{k_{ee}}=0.5$, $\sqrt{k_{ei}}=1.5$, $\sqrt{k_{ie}}=1$, $\sqrt{k_{ii}}=2$, *K* = 60, *N* = 1000. To improve the accuracy of the theory to finite *K*, *N*, here we use a slightly modified connection weight, $w_{0}/\sqrt{K_{\alpha \beta }\left(1-K_{\alpha \beta }/N\right)}$, for all excitatory non-zero connections, and similarly $-w_{0}/\sqrt{K_{\alpha \beta }\left(1-K_{\alpha \beta }/N\right)}$ for inhibitory connections, such that $\text{var}\left(J_{ij}\right)=w_{0}^{2}/N$ holds exactly for finite *N*. **B**. Similar as A but for balanced EI network (see text) with *k*_*αβ*_ = *k* = 1, *g* = 0.4, *K* = 40, *N* = 400. Note there are two outliers on both sides of the bulk.

To map this architecture on to the one studied above, we adopt the framework of [21] and consider the equivalent Gaussian connectivity with matching variance for each *J*_*ij*_. Importantly, the choice of connection probabilities and weights ensures that var(*J*_*ij*_) is $w_{0}^{2}/N$ (to the leading order for *N* ≫ 1) regardless of the cell type of neuron *i*, *j*. This allows us to define the effective synaptic gain as $g^{2}=w_{0}^{2}$ for all neurons. The mean of the connections between a presynaptic neuron of type *β* and postsynaptic *α* is $E(J_{ij})=\sqrt{K_{\alpha \beta }}w_{\beta }/N$ where *w*_*E*_ = *w*_0_ and *w*_*I*_ = −*w*_0_. Thus, we can write *J*_*ij*_ as a zero-mean i.i.d. Gaussian matrix with uniform variance $w_{0}^{2}/N$ and a rank-2 matrix of the means. As stated in Section 3.4, in such a case the bulk spectrum of the neurons’ covariance matrix is the same as Eq (5). In addition there are at most 4 outlier eigenvalues. For *K* ≫ 1, from the analysis of [21], the stability of the recurrent dynamics of a linear network with the above connectivity amounts to the requirement that all eigenvalues of the 2 × 2 matrix $M_{\alpha \beta }=\sqrt{k_{\alpha \beta }}w_{\beta }$ have negative real parts. Fulfilling this condition by choosing appropriate values for *k*_*αβ*_ (see example in Fig 6A and Section C.3 in S1 Text) guarantees that the outlier(s) due to the nonzero means are to the left of the bulk covariance spectrum so that the largest eigenvalue is *x*_+_(*g*), Eq (6). For *K* = *O*(1), the results in [43] show that the above condition is sufficient but not necessary for stability. For example, when all *k*_*αβ*_ are equal to *k*, which corresponds to a balance of excitation and inhibition [45], all eigenvalues of *M* are 0 and the dynamics is stable for *g* < 1 for large *N*. At the same time, there can be two outlying eigenvalues on the two sides of the bulk covariance spectrum (Fig 6B), whose expected location can be predicted (Section 3.4 and Section C.3 in S1 Text). Several additional examples including all inhibitory networks are considered in the Section F in S1 Text.

### 3.6 Frequency dependent covariance

We have so far focused on the long time window covariance matrix. This would be especially suitable for neural activity recordings with limited temporal resolution such as calcium imaging [46]. Temporal structures of correlation beyond the slow time scale can be described by the frequency covariance matrix (or coherence matrix)

$$\begin{array}{c} C_{ij}\left(\omega \right)=\underset{\Delta T\to \infty }{\text{lim}}\left\langle \Delta s_{i}\left(\omega \right)\Delta s_{j}^{†}\left(\omega \right)\right\rangle , \end{array}$$ (19)

where $\Delta s_{i}\left(\omega \right)=\frac{1}{\sqrt{\Delta T}}\int_{0}^{\Delta T}\Delta e^{-i\omega }x_{i}\left(t\right)dt$ is the Fourier transform of the neural activity and *z*^†^ is the complex conjugate. *C*_*ij*_(*ω*) can also be calculated by the Fourier transform of the time-lagged cross-correlation functions *C*_*ij*_(*τ*) = 〈*x*_*i*_(*t*)*x*_*j*_(*t* − *τ*)〉 (Wiener-Khinchin theorem). Analogous to Eq (2)*C*(*ω*) obeys [25],

$$\begin{array}{c} C\left(\omega \right)=\sigma^{2}{\left|a\left(\omega \right)\right|}^{2}{\left(I-a(\omega )J\right)}^{-1}{\left(I-a^{†}\left(\omega \right)J\right)}^{-T}. \end{array}$$ (20)

Here *z*^†^ is the complex conjugate and |*z*| is the norm for a complex *z*. The transfer function *a*(*ω*) = (1 + *iτω*)^−1^ summarizes the dynamics of single neurons in the network and corresponds to a response filter of *e*^−*t*/*τ*^/*τ*, *t* > 0 for the model of Eq (1) (see also Section 5.2). The long time window covariance we have studied corresponds to *C*(*ω* = 0) (Wiener-Khinchin theorem).

For the i.i.d. Gaussian random connectivity *J*, we can show that the spectrum of *C*(*ω*) is given by the same Eq (5) for *C*(0) (up to a constant scaling) by replacing *g* with a frequency dependent *g*(*ω*) (compare with Eq (3))

$$\begin{array}{c} g\left(\omega \right)=\left|a\left(\omega \right)\right|\sqrt{N\;\text{var}(J_{ij})}=\frac{g}{\sqrt{1+\tau^{2}\omega^{2}}}. \end{array}$$ (21)

We can use Eq (21) to study the scaling of frequency as *g* approaches the critical value of 1. In many cases, we can expect that the neuronal and synaptic dynamics lead to a smooth effective low-pass filtering of the recurrent input, such that for small frequency *g*(0) − *g*(*ω*) ∝ *ω*^2^. For the specific *g*(*ω*) in Eq (21), this can be directly verified. The low-pass filtering implies that the frequencies showing a critical covariance spectrum are those with $\left|\omega |=o({\left(1-g\right)}^{\frac{1}{2}}\right)$.

Note, however, the simple replacement by an effective *g* may not apply to a connectivity matrix that does not have i.i.d. entries. For example, for networks with non-zero reciprocal motifs, the covariance spectrum changes qualitatively with frequency (Section D.1 in S1 Text).

### 3.7 Sampling in time and space

The theoretical spectra we have discussed are based on the exact covariance matrix (Eq (2)). For neural data, this is equivalent to the limit of the sample covariance $\hat{C}$ (Eq (37)) when the number of time samples *M* is much larger than the number of neurons *N*. Note that if the activity data is first averaged or summed over a time window/bin (Δ*T* in Eq (37)) before calculating the sample covariance, then *M* is the number of bins. However, many large-scale neural recordings are in the so-called *high dimensional* regime, where *N* and *M* are comparable, that is, the ratio *α* = *N*/*M* remains finite or even greater than 1 for large *N*, *M*. It is thus important to study this effect of temporal sampling on the covariance eigenvalues to better relate to experimental data [7].

We refer to $\hat{C}$ and $p_{\hat{C}}\left(x\right)$ as the *time-sampled* covariance and spectrum. The relation between the original spectrum *p*_*C*_(*x*) and the time-sampled spectrum $p_{\hat{C}}\left(x\right)$ for a finite *α* ≥ 0 has been studied in [47]. The authors derived a general relation between the generating function of the eigenvalue distribution $W\left(z\right)=\sum_{n=1}^{\infty }z^{n}\mu_{n}$, where *μ*_*n*_ is the *n*-th moments of the eigenvalue distribution, and the counterpart $\hat{W}\left(z\right)$ for the sampled distribution,

$$\begin{array}{c} \hat{W}\left(z\cdot \left(1+\alpha W\left(z\right)\right)\right)=W\left(z\right),\;\text{and}\;\text{conversely}\;\;\;W(\frac{z}{1+\alpha \hat{W}\left(z\right)})=\hat{W}\left(z\right). \end{array}$$ (22)

We give an alternative derivation of this result using free probability [48–50] (Section H in S1 Text), which allows us to also generalize to the spatial sampling case (see below). For simplicity, here we describe the results for 0 ≤ *α* ≤ 1. For *α* > 1 where time samples are severely limited, the spectrum of the *N* − *M* nonzero eigenvalues can be calculated with small modifications (Section H.2 in S1 Text).

One corollary of Eq (22) is a simple formula for how the (relative) dimension changes under time sampling

$$\begin{array}{c} D\left(\hat{C}\right)=D\left(C\right)\frac{N}{N+\alpha D(C)},\;\hat{D}\left(\hat{C}\right)=\frac{\hat{D}\left(C\right)}{1+\alpha \hat{D}\left(C\right)}. \end{array}$$ (23)

These formulas show that both *D* and $\hat{D}=D/N$ decrease with *α* (fewer time samples).

**Fig 7:**
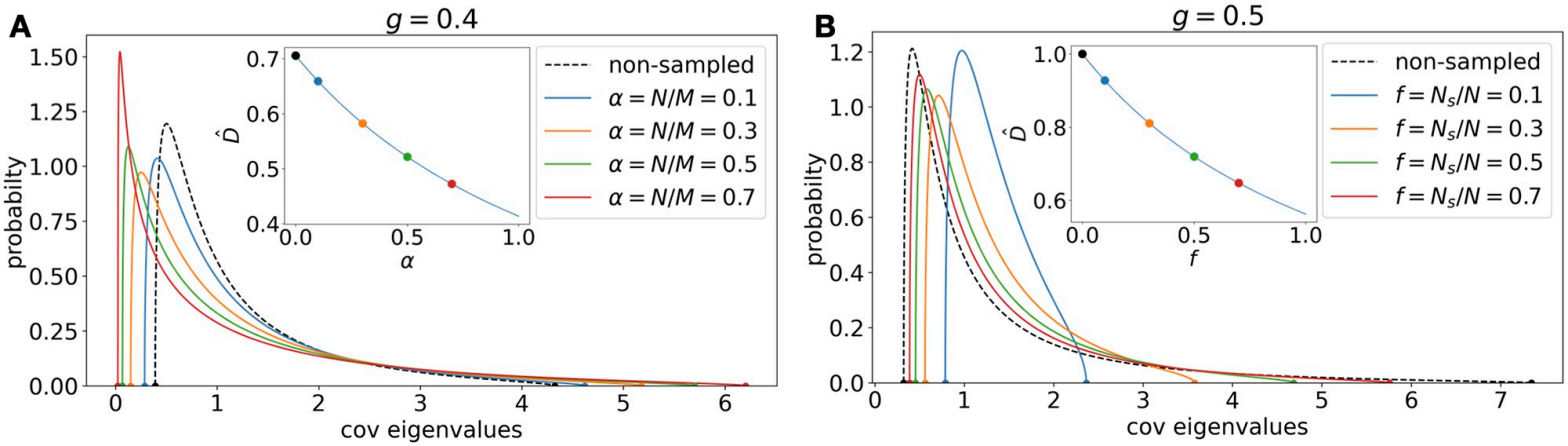
Effects of sampling in time and space on the covariance spectrum. **A**. For the i.i.d. Gaussian random connectivity, how different levels of time samples *α* change the spectrum (Eq (22)). The non-sampled case corresponds to *α* = 0. *g* is fixed at 0.4. Inset: The relative dimension $\hat{D}$ vs. *α* (Eq 23). The dots correspond to the pdfs with matched colors. **B**. Same as A. but for the spatial subsampling (Section I.2 in S1 Text), at *g* = 0.5. The non-sampled case corresponds to *f* = 1. The relative dimension in the inset is based on Section I.1 in S1 Text.

The relations Eqs (22) to (23) apply to any covariance matrix spectrum. For example, it reproduces the time-sampled dimension derived in [7] for a different model of covariance *C*. We now apply Eq 22 to the case of i.i.d. Gaussian connectivity to derive specific results of the time-sampled spectrum $p_{\hat{C}}\left(x\right)≕p_{g,\alpha }\left(x\right)$. Here Eq (22) becomes a cubic equation and can be solved analytically (Section H.3 in S1 Text). Consistent with the dimension, when *α* increases from 0 to 1, the support of the time-sampled distribution expands from both sides (Fig 7A). In particular, for any fixed *α* < 1 (so $\hat{C}$ is positive definite), the left edge of the support *x*_−_ decreases with *g* but is always bounded away from 0 even as *g* → 1, where $x_{-}\to \frac{2}{27}({\left(1+3\alpha \right)}^{\frac{3}{2}}+1-9\alpha ))$ (see also Fig L in S1 Text). Interestingly, the approximate power law of *p*_*C*_(*x*) (Eq 7) still holds under time sampling for any fixed *α* as *g* → 1 (Section H.3 in S1 Text).

Another challenge for fitting to neural data is that often only a subset of neurons are observed instead of the entire local recurrent network. The unobserved neurons have an impact on the dynamics and affect the eigenvalues of the observed covariance matrix. We study this by considering randomly selecting *N*_*s*_ = *fN*, 0 < *f* ≤ 1 neurons and define their covariance $\tilde{C}$ as the *space-sampled* covariance. Using the free probability approach, we derive similar results as Eqs (22) and (23) (Section I in S1 Text) and apply them to derive the spectrum and dimension for the i.i.d. Gaussian connectivity under spatial sampling. In particular, the relative dimension $\hat{D}\left(\tilde{C}\right)=D\left(\tilde{C}\right)/\left(fN\right)$ increases with spatial sampling (i.e., decrease *f*) which is consistent with the shape of the spectrum where its support narrows in (Fig 7). Lastly, the power-law feature is also preserved under spatial sampling. For any fixed 0 < *f* ≤ 1, we show that as *g* → 1 and *x* → ∞ (see examples with *g* close to 1 in Fig L in S1 Text)

$$\begin{array}{c} p_{g,f}\left(x\right)\approx \frac{\sqrt{3}}{2\pi }f^{-\frac{1}{3}}x^{-\frac{5}{3}}. \end{array}$$ (24)

### 3.8 Fitting the theoretical spectrum to data

Our theory for the bulk covariance spectrum can be fitted to neural activity whenever the covariance eigenvalues can be calculated. The best-fitting theoretical spectrum can be found by minimizing the *L*^2^ or *L*^∞^ error between the empirical and theoretical cumulative distributions (Methods) with respect to parameters such as *g*. We note that the availability of closed-form or analytic solutions of the theoretical distributions makes this optimization highly efficient.

In many settings, the value of the baseline neuronal variance *σ*^2^ in Eq (2) is not known. But this can be easily addressed by scaling both the observed and the predicted eigenvalues to have a mean equal to 1. After fitting the connectivity parameter *g* for the normalized eigenvalues, *σ*^2^ can then be estimated using the original means of data and theory. For our theoretical spectra, the mean *μ* of covariance eigenvalues is available in closed-form (Eqs (9) and (18)), and the scaled pdf is easily found as *p*_*R*_(*x*) = *μp*_*C*_(*μx*).

Furthermore, the recorded neural activity is sometimes normalized for each neuron (e.g., by converting activity to z-scores). In this case, we need to analyze the eigenvalues of a *correlation matrix* whose entries are normalized as $C_{ij}/\sqrt{C_{ii}C_{jj}}$. Interestingly, we found that the correlation eigenvalue distribution for our random connectivity models in the large *N* limit is the same as the rescaled *p*_*R*_(*x*) above. This is because the diagonal entries of *C* become uniform (thus converge to *μ*) for large *N* (Section J in S1 Text).

The fitting of the spectrum is also robust to outliers in the covariance eigenvalues (Section 3.4). In Section K in S1 Text we demonstrate an example where a rank-2 component is added to the covariance *C*. Since in practice the rank of the perturbation is unknown *a priori*, we use *all* eigenvalues in the fitting, and the fitted *g* is highly accurate despite the presence of outliers. We can also use the fitted *g* to help identify the outliers by separating them out based on the upper edge of *p*_*C*_(*x*) support [32, 51].

**Fig 8:**
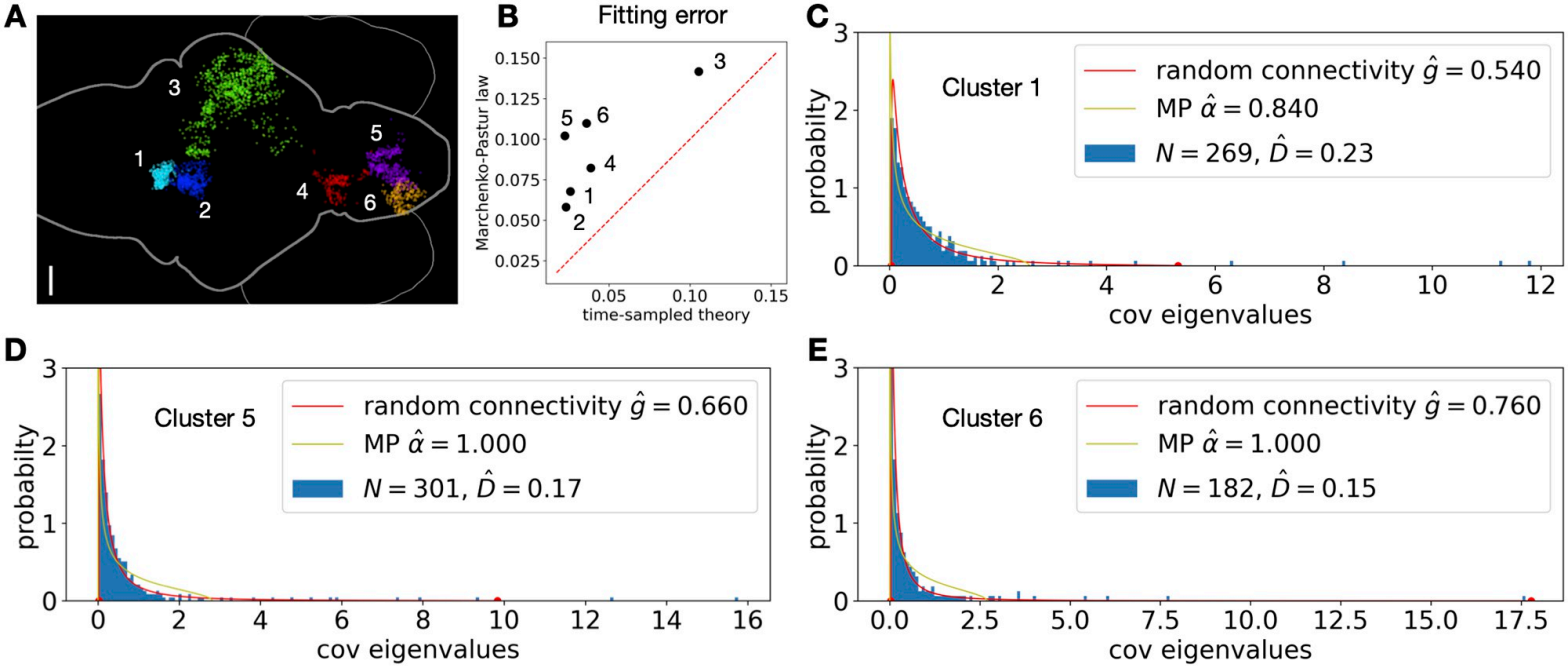
Fitting the theoretical spectrum to data. **A**. The anatomical map of neurons (dots) in the example functional clusters (different colors) across a larval zebrafish brain (scale bar is 50 *μ*m, see text and [52]). **B**. Comparing the fitting error of the time-sampled random connectivity theory (Section 3.7) and the Marchenko–Pastur law. The errors are measured by Eq (38). The red dashed line is the diagonal. $\hat{D}=D/N$ labeled on each plot is the relative dimensionality (Eq 4). The calcium activity is recorded at a frame rate of 2 Hz and a total of 600 frames of spontaneous activity [52] are used in here. See more details in Methods. Fitting results for all other clusters are in Fig O in S1 Text.

We conclude with a preliminary application to whole-brain calcium imaging data in larval zebrafish. In [52], activities of the majority of the neurons in the larval zebrafish brain were imaged simultaneously during presentations of various visual stimuli and grouped into *functional clusters* based on their response similarity. These clusters reveal potential neural circuits and, in some cases, reveal a good match with known brain nuclei. Here we select a few clusters that contain a large number of neurons and are anatomically localized (Fig 8B). For each cluster, we calculate its sample correlation matrix during the spontaneous condition (no stimulus was presented) and then fitted the eigenvalues to the time-sampled spectrum with i.i.d. Gaussian connectivity (Section 3.7). Here the calcium activity is used but the correlation matrix is expected to be approximately the same if firing rates or long time window spike counts were used (Section K in S1 Text). Despite the simplicity of the model with only one parameter to tune, the results show good agreement with data and is significantly better than fitting using the Marchenko–Pastur law (Fig 8C), which models spatially independent noise (Section 5.4). Therefore, our theory provides a quantitative mechanistic explanation of how a long tail of covariance eigenvalues or equivalently low dimensional activity occurs in recurrent neural circuits.

## 4 Discussion

In this work, we studied the eigenvalue density distribution of the covariance matrix of a randomly connected neuronal network, whose activity is approximated as noise driven linear fluctuations around a steady state. We derived an explicit expression for eigenvalue distribution in the large-network limit analytically in terms of the statistics of the connectivity such as coupling strength and second-order motifs. Our results also include closed-form expressions for the dimension measure generalizing known results [6]. Some of these dimensionality results are also derived in a recent parallel work [37], whereas the shape of the covariance spectrum was not studied in [37]. Knowing the exact shape and support of the bulk spectrum can facilitate separating outlying eigenvalues corresponding to low dimensional structure (coming from other unmodeled effects such as external input) from variability due to noise [51] (see an example in Fig N in S1 Text). The shape of the bulk spectrum reflects structured amplification of the neuronal noise by the random recurrent interactions. Since the bulk spectrum is not altered by low rank perturbations to the connectivity or to the activity (Section C in S1 Text), this allows for distinguishing different sources of variability in neural data. As the connection strength increases towards the critical edge of stability, the spectrum exhibits a power-law tail of large eigenvalues, with exponent −5/3 in pdf (or −3/2 in eigenvalue vs. rank plot). This power-law shape near the critical *g* provides concise theoretical characterizations of the spectra under various connectivities. Intriguingly, the same power law persists even when the shape of the spectrum is modified by connectivity motifs or due to finite temporal and spatial sample size. In contrast, when we move away from the asymmetric, random connectivity model, the exponent of the power law (if any) becomes different: −7/4 for symmetric random connectivity (Eq (16)), −2 for a normal connectivity *J*^*n*^ with matching eigenvalue distribution as i.i.d. Gaussian *J* (Section E.3 in S1 Text), and −*d*/4 − 1 for a *d*-dimensional ring network (see below). Based on these results, we conjecture that a power-law tail, whenever present for any covariance spectrum, reflects the qualitative nature of the connectivity and is a robust feature that will survive both temporal and spatial sampling with the same exponent (precise statement in Section I.3 in S1 Text).

Unlike the large eigenvalues [53], the interpretation of the bulk spectrum of PCA of neural activity data has received little attention. A notable exception is a recent work [54] which studied the power law of covariance spectrum of data near criticality based on the renormalization group method. By fitting experimental data to the theoretical spectrum, information from eigenvalues of all sizes can be used to estimate the effective connection strength (Fig 8). Our theory thus provides an important benchmark to compare with experimental data and advocates the bulk covariance spectrum as a powerful global description of collective dynamics in neuronal networks.

### 4.1 Nonlinear dynamics

One limitation of the work is the assumed dynamic regime where fluctuations of the neuronal activity are described by the linear response theory [22, 23] around a fixed point. While extending the theory to highly nonlinear activity such as chaotic dynamics [2] is left for future research, we provide some numerical examples of networks with nonlinear neurons to illustrate the applicability of our results.

**Fig 9:**
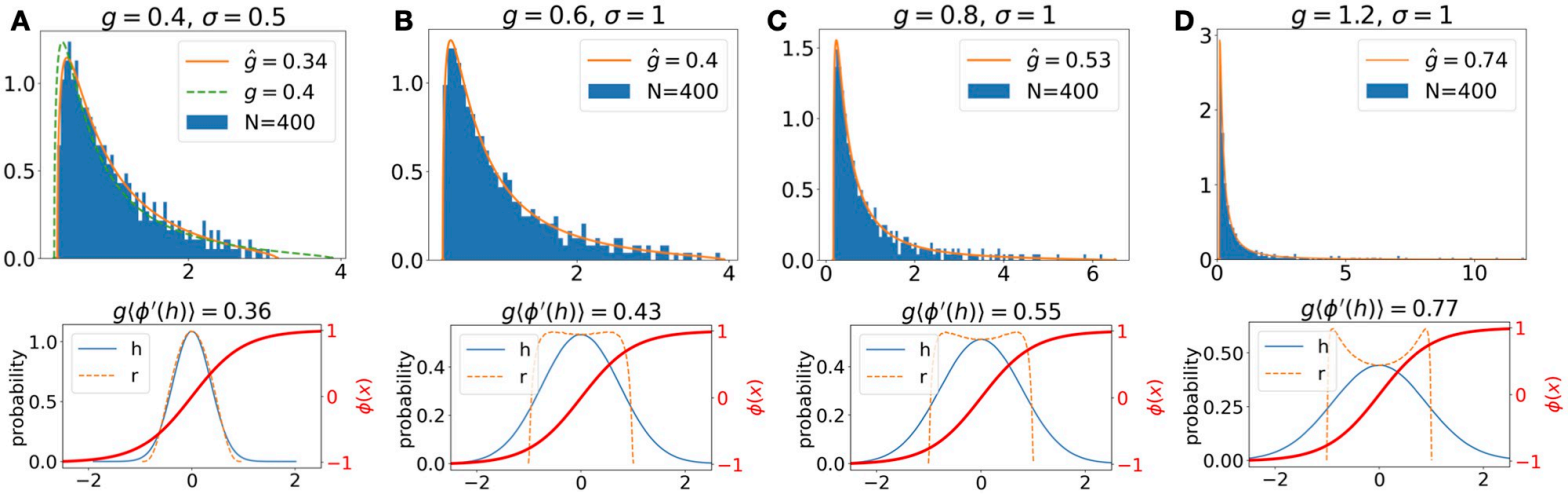
Covariance spectrum in nonlinear dynamics. **A.** Top: A histogram of covariance eigenvalues calculated from firing rate activities *r*_*i*_(*t*) of simulating a *N* = 400 network model according to Eq 33. The eigenvalues are normalized to have a mean equal to 1 for easy comparison of the shape (or equivalently the eigenvalues of the correlation matrix Section 3.8). Here *g* = 0.4 and *σ* = 0.5 (see Methods for additional numerical details). The green dashed line is the time-sampled theoretical spectrum (Section 3.7) using actual *g* and *α*, only shown in A for clarity of the plots. The length of the simulated data corresponds to *α* = *N*/*T* = 0.1. The orange curve is also the time-sampled theoretical spectrum except for using an effective $\hat{g}$ that is fitted numerically to best match the simulated eigenvalues. Bottom: The blue curve is the histogram of *h*_*i*_(*t*) (aggregated across *i* and *t*) and the orange dashed curve is the histogram of *r*_*i*_(*t*). The overlaying red curve shows the nonlinearity *ϕ*(*x*) as a reference. The 〈⋅〉 in the title of each plot represents averaging *ϕ*′(*h*_*i*_(*t*)) over all *i* and *t*. **B-D**. Same as A except for *σ* = 1 and *g* = 0.6, 0.8, 1.2, respectively. Only the fitted theoretical spectrum (orange curve) is shown for clarity.

We consider a model with nonlinear rate neurons driven by external noise by introducing an activation function *ϕ*(*x*) = tanh(*x*) in Eq (1) [2] [55]. The nonlinearity transforms the currents *h*_*i*_(*t*) to firing rates *r*_*i*_(*t*) = *ϕ*(*h*_*i*_(*t*)), and the recurrent interaction term is now $\sum_{j=1}^{N}J_{ij}r_{i}\left(t\right)$ (Eq (33) in Methods). When the connection strength *g* = Nvar(*J*_*ij*_) and the noise magnitude *σ* are small, the neural activity will mainly fluctuate near 0 where *ϕ*(*x*) can be approximated by linearizing around 0 (Fig 9A, bottom). Indeed, the spectrum based on the linear theory (green dashed line in Fig 9A, top) approximates the numerical spectrum. For larger *g* and *σ* the deviation from the linear theory becomes significant as the firing rates are now strongly shaped by the nonlinearity (Fig 9B, 9C and 9D, bottom). Interestingly, these spectra can be well fitted by the linear-theory spectrum if *g* is replaced by a smaller effective $\hat{g}$. This reduction in the effective *g* can be qualitatively understood as the average slope of *ϕ*(*x*) over the distribution of *h*_*i*_(*t*) (Fig 9 bottom). Note that, unlike the noiseless model in [2], the cases studied here are not chaotic even with *g* > 1. As shown in [55], the presence of external noise shifts the transition to chaotic dynamics from *g* = 1 to larger values. A theoretical characterization of effective *g* and other strongly nonlinear dynamics such as chaotic dynamics is left for future research. Future work could also consider cases with transient activity which are common in nonlinear systems and more general network architectures, such as multiple populations of EI networks [21] and incorporating distant dependent connectivity patterns based on known cortical microcircuit architectures [56–58].

### 4.2 Ordered vs. disorder connectivity

We have studied the covariance spectrum under random connectivity, which is used as a model for complex recurrent networks. Here we ask whether features of these spectra are distinct results of the connectivity being random. To address this, we briefly explored the covariance spectra from several widely used examples of ordered connectivity for comparison.

**Fig 10:**
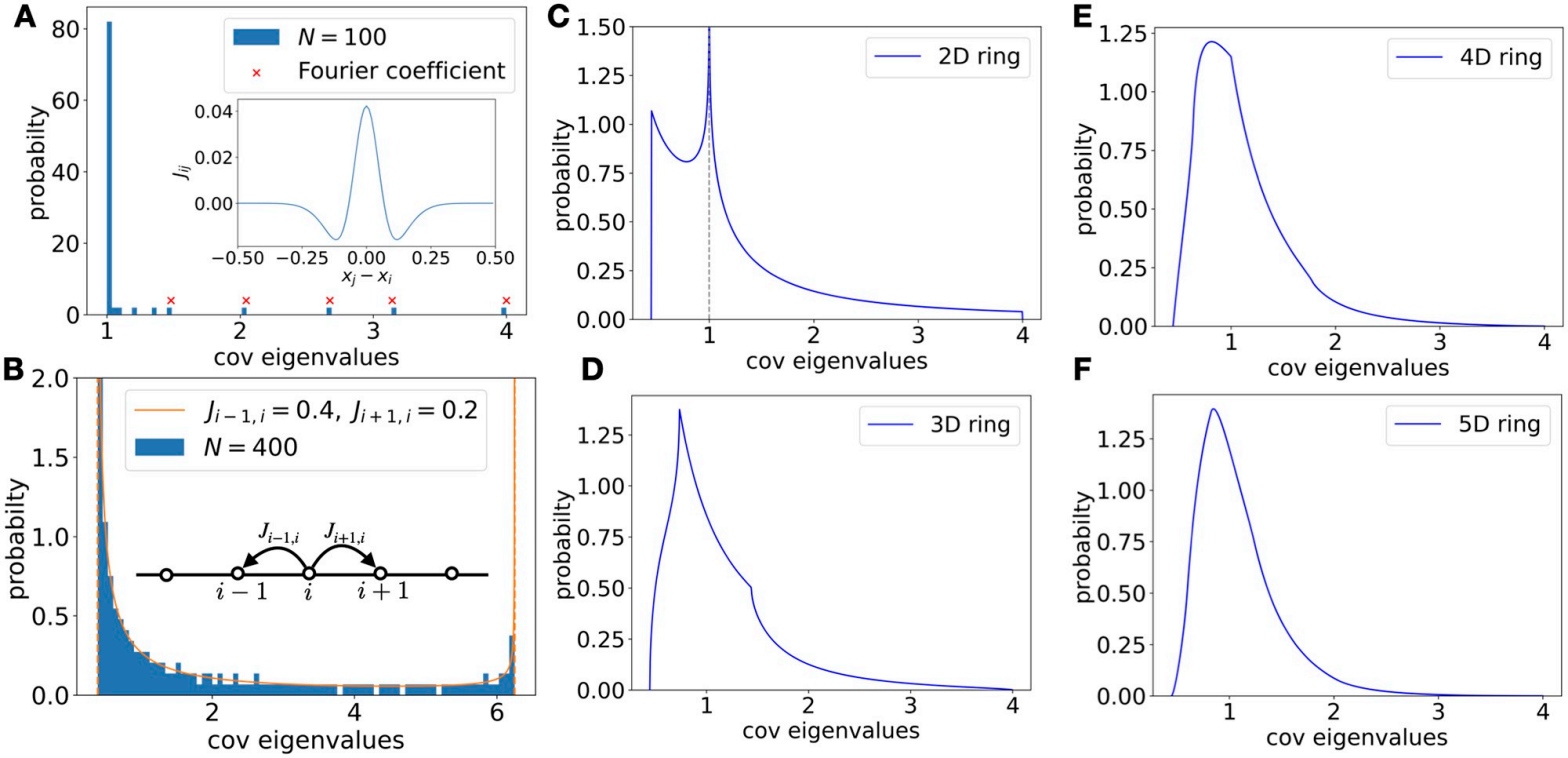
Covariance spectra under some deterministic connectivity models. **A**. Histogram of the covariance eigenvalues of a ring network with a long-range connection profile (inset, *N* = 100). Most eigenvalues are close to 1 and the rest of eigenvalues converge to discrete locations predicted by top Fourier coefficients (crosses) of the connection profile (Eq (36)). **B**. Same as A. but for a ring network with Nearest-Neighbor connections: *J*_*i*−1_ = 0.4, *J*_*i*+1,*i*_ = 0.2. The solid line is theoretical spectrum in large *N* limit which has two diverging singularities at both support edges. The effect of such singularities is also evident in the finite-size network at *N* = 400 (a single realization). **C-F**. Higher dimensional Nearest-Neighbor ring network (*ad* = 0.6, see Methods). As the dimension increases, the singularities in the pdf become milder and less evident, and the overall shape becomes qualitatively similar to the random connectivity case (Fig 1).

First, consider a ring network [56] with translation invariant long-range connections, where the connection strength between neurons depends smoothly on their distance (Fig 10A inset, see Methods). In the large-network limit, the covariance spectrum becomes a delta distribution at 1 with a few discretely located large eigenvalues (Fig 10A). Next, we consider the ring network with short-range, in particular, Nearest-Neighbor (NN) connections. The covariance spectrum is now continuous (no outliers) and supported on an interval, but the pdf diverges at both edges as ${\left(\Delta x\right)}^{-\frac{1}{2}}$ (Fig 10B).

To seek further examples of ordered connectivity leading to a qualitatively similar covariance spectrum as the random connectivity, we consider the *d*-dimensional generalization of the NN ring (Methods). As dimension *d* increases, the smoothness of the pdf within and at the edges of the support increases, and the covariance spectrum becomes qualitatively similar to the random case [59] (Fig 10). Interestingly, as the connection strength approaches its critical value for stability, the covariance spectra also exhibit a power-law tail $p_{C}\left(x\right)\propto x^{-\frac{d}{4}-1}$ (Section L.3.1 in S1 Text; $x^{-\frac{d}{2}-1}$ is also possible under other cases using [60]). To match the exponent of the random network *d* would be 8/3 ≈ 2.67. These comparisons suggest that the covariance spectrum’s overall smooth density and long tail shape is a shared property in highly connected networks with high rank connectivity matrices, including random networks and high dimensional short-range spatially invariant networks.

## 5 Methods

### 5.1 Models of random connectivity

Here is a summary of results on various random connectivity models.

- i.i.d. Gaussian random connectivity $J_{ij}∼N\left(0,g^{2}/N\right)$: closed-form pdf and endpoints (Eq (5)), including the frequency-dependent covariance spectrum (Section 3.6), and a power-law tail approximation (Eq (7)).
- Gaussian random connectivity with reciprocal motifs/asymmetry *κ* = *ρ*(*J*_*ij*_, *J*_*ji*_) (Section 3.3): analytic solution and endpoints (quartic root, Section E in S1 Text) and a power-law tail approximation (Eq 17). For special case of symmetric and ant-symmetric connectivity, closed-form pdf Eqs (14) and (15), including a frequency dependent covariance spectrum (Section G in S1 Text).
- Erdős-Rényi and certain EI network Section 3.5: same bulk spectrum as the i.i.d. Gaussian case.

For all cases, the mean *μ* and the dimension *D* are derived in closed-form (Eqs 9, (10) and (18).

For simplicity, we do not require *J*_*ii*_ to be zero (i.e., no self-coupling), but allow it, for example in the i.i.d. Gaussian model, to be distributed in the same way as other entries *J*_*ij*_. In the large-network limit, since individual connections are weak (e.g., $O(1/\sqrt{N})$), allowing this self-coupling or setting *J*_*ii*_ = 0 does not affect the covariance spectrum (Section A.3 in S1 Text).

### 5.2 Applications to alternative neuronal models and signal covariance

Although the relation between *C* and *J* (Eq (2)) is derived here in a linear rate neuron network Eq (1), it also arises in other models of networked systems.

#### Linearly interacting Poisson neurons

This is also called a multivariate Hawkes model [27]. This is a simple model for spiking neuron networks, but is versatile enough to capture for example the temporal spiking correlations seen in other more sophisticated nonlinear spiking neuron networks [26, 28]. A time-dependent Poisson firing rate is calculated as a filtered input spike train *s*_*j*_(*t*) (sum of delta functions), and spikes are then drawn as a Poisson process given *y*_*i*_(*t*),

$$\begin{array}{c} y_{i}\left(t\right)=y_{0}+\int_{0}^{\infty }A\left(t-\tau \right)(\sum_{j}W_{ij}s_{j}\left(t-\tau \right))d\tau . \end{array}$$ (25)

Here we consider a homogeneous network where the baseline firing rate *y*_0_ and response filter *A*(*t*) is the same for all neurons.

The exact long time window spike count covariance matrix of this network can be shown to be [27]

$$\begin{array}{c} C={\left(I-aW\right)}^{-1}C^{0}{\left(I-aW\right)}^{-T},\;C^{0}=\text{diag}\left\{Y_{1},Y_{2},,\ldots ,Y_{N}\right\},\;Y={\left(I-aW\right)}^{-1}Y^{0}, \end{array}$$ (26)

which is valid if the time varying *y*_*i*_(*t*) does not often become negative (for example when any negative connections *W*_*ij*_ are small compare to *y*_0_). Here $a=\int_{0}^{\infty }A\left(t\right)dt$, *Y*^0^ and *Y* are vectors of baseline and perturbed (with recurrent connections) firing rates of the neurons respectively. If we assume that the effective connection strength *aW* is weak so that we can approximate *Y* with *Y*^0^, then (26) becomes

$$\begin{array}{c} C=y_{0}{\left(I-aW\right)}^{-1}{\left(I-aW\right)}^{-T}, \end{array}$$

the same as Eq (2) with *J* = *aW* (note that for Poisson process $\text{var}\left(\int_{t}^{t+\Delta t}s_{i}\left(u\right)du\right)=\int_{t}^{t+\Delta t}y_{i}\left(u\right)du$).

Another condition that ensures a uniform *Y* and does not restrict connections to be weak is a *row balance* condition of *W* sometimes assumed in EI networks [45],

$$\begin{array}{c} \sum_{j=1}^{N}J_{i_{1}j}=\sum_{j=1}^{N}J_{i_{2}j}. \end{array}$$ (27)

This is not unreasonable to assume, for example, considering the homeostatic mechanisms of neurons.

#### Integrate-and-Fire neurons

As shown in [23, 26] the linear response theory [22] can be used to approximately describe the covariance structure of a network of generalized integrate-and-fire (IF) neurons

$$\begin{array}{c} \tau \frac{dV_{i}}{dt}=-\left(V_{i}-E_{L,i}\right)+\psi \left(V_{i}\right)+E_{i}+\sqrt{\sigma_{i}^{2}\tau_{i}}\xi_{i}\left(t\right)+\sum_{j}W_{ij}F_{ij}\left(t\right)*y_{j}\left(t\right). \end{array}$$ (28)

Here *V*_*i*_ is the membrane potential and a spike is generated when *V*_*i*_ reaches a threshold. *y*_*i*_(*t*) = ∑_*k*_* δ*(*t* − *t*_*i*,*k*_) is the spike train, and $y_{i}^{0}\left(t\right)$ in Eq (29) is the “unperturbed” spike train in absence of recurrent connections *W*. Different choices of *ψ*(*V*) realize types of IF neurons, such as *ψ*(*V*) = Δ_*T*_ exp((*V* − *V*_*T*_)/Δ_*T*_) for the exponential IF neurons. During the asynchronous firing of neurons (no strong synchronized firing across the network), Eq (28) can be well approximated by

$$\begin{array}{c} \Delta y_{i}\left(t\right)=A_{i}\left(t\right)*(\sum_{j=1}^{N}W_{ij}F_{ij}\left(t\right)*\Delta y_{j}\left(t\right))+\Delta y_{i}^{0}\left(t\right),\;i=1,2,\ldots ,N. \end{array}$$ (29)

Here $a\left(t\right)*b\left(t\right)=\left(a*b\right)\left(t\right)=\int_{0}^{t}a\left(s\right)b\left(t-s\right)ds$ denotes convolution. *W* = {*W*_*ij*_} is the matrix of recurrent connection weights. *A*_*i*_(*t*) is the linear response kernel for neuron *i* (e.g., an exponential decay) *F*_*ij*_(*t*) is the temporal kernel of the synapse. For simplicity, we assume that both *A* and *F* are uniform across the network.

It it shown [23, 26] that the long time window spike count covariance matrix *C* (in fact also the frequency covariance Eq (19)) is well approximated by

$$\begin{array}{c} C=a^{2}\left\langle {\left(\Delta y_{i}^{0}\left(t\right)\right)}^{2}\right\rangle {\left(I-aW\right)}^{-1}{\left(I-aW\right)}^{-T}. \end{array}$$

Here the scalar $a=(\int_{0}^{\infty }A\left(t\right)dt)(\int_{0}^{\infty }F\left(t\right)dt)$ summarizes the cellular and synaptic dynamics. $\langle {\left(\Delta y_{i}^{0}\left(t\right)\right)}^{2}\rangle$ can be thought of as the baseline neuronal variance in the absence of recurrent connections (*A* = 0 in Eq (29). This expression of the covariance matrix again matches with Eq 2.

#### Fixed points over whitened input

The covariance we considered so far describes the structure of fluctuations of spontaneous dynamics without or under fixed external input, often referred as the *noise covariance* [61]. We can also consider the spectrum for a *signal covariance*. This perspective is needed to use our results to interpret experimental data where the neural activity is largely driven by stimuli for example [10].

Consider a network of linear firing rate neurons,

$$\begin{array}{c} \tau \frac{dr_{i}}{dt}=-r_{i}+\sum_{j=1}^{N}J_{ij}r_{j}+u_{i},\;i=1,2,\ldots ,N. \end{array}$$ (30)

Here *u*_*i*_ is the external input to neuron *i*. Assume the network settles to a steady state, so the fixed point firing rates are given by

$$\begin{array}{c} \vec{r}={\left(I-J\right)}^{-1}\vec{u}. \end{array}$$ (31)

Now consider the network activity across an ensemble of input patterns, which has whitened statistics [62],

$$\begin{array}{c} \text{var}\left(u_{i}\right)=\sigma^{2},\;\text{cov}\left(u_{i},u_{j}\right)=0. \end{array}$$ (32)

It is easy to see that the covariance of firing rates $\vec{r}$ is given by *σ*^2^(*I* − *J*)^−1^(*I* − *J*)^−*T*^, which is the same as Eq (2).

We note that Eq (31) or equivalently $\vec{r}=J\vec{r}+\vec{u}$ appears in broader contexts beyond neuroscience and is studied in the field of linear structural equation modeling (SEM) [29].

#### Nonlinear rate neurons

The following is a simple and classic nonlinear rate neuron model similar to that used in [2],

$$\begin{array}{c} \tau {\dot{h}}_{i}\left(t\right)=-h_{i}\left(t\right)+\sum_{j=1}^{N}J_{ij}\phi \left(h_{j}\left(t\right)\right)+\xi_{i}\left(t\right),\;i=1,\ldots ,N. \end{array}$$ (33)

Here *ϕ*(*x*) = tanh(*x*) converts currents *h*_*i*_(*t*) into firing rates *r*_*i*_(*t*) = *ϕ*(*h*_*i*_(*t*)). The white noise *ξ*_*i*_ and i.i.d. Gaussian random *J* are the same as in Eqs (1) and (3). The presence of noise allows for nontrivial activity for *g* < 1. Due to symmetry of *ϕ*(*x*) and *J* distribution, the average activity over time 〈*h*_*i*_(*t*)〉 = 0 for large *N*. Note that the largest slope of *ϕ*(*x*) is 1 at *x* = 0, our main model Eq (1) can be viewed as a linear approximation to Eq (33) and the definition of connection strength *g* is consistent.

In Fig 9, *N* = 400, *τ* = 1 and Eq (33) is simulated using the Euler-Maruyama method with a time step of 0.01 for a duration of *T*_0_ = 40000. To get the long time window covariance, the firing rates *r*_*i*_(*t*) are binned by a time window of 10, which is sufficient based on examining the decay of its autocorrelation function. After binning, the simulated data correspond to a time-sample parameter *α* = *N*/*T* = 0.1 (3.7) and this *α* is used in calculating the theoretical spectra. The empirical covariance matrix is then calculated from the binned firing rates according to Eq (37). As shown in Fig 9, the theoretical spectrum based on the linear dynamics can still be used to fit the simulated spectrum in this nonlinear network when *g* is replaced by a smaller effective $\hat{g}$.

### 5.3 Power-law approximation of the eigenvalue distribution

The power-law property of *p*_*C*_(*x*) for i.i.d. *J*_*ij*_ under critical *g* is probably known in random matrix theory (private communication), by results from the equivalent distribution studied in [30], although we do not know of a specific reference. We include a derivation based on the explicit expression of *p*_*C*_(*x*) (Eq (5)) that is outlined below.

First note the limits of the support edges. As *g* → 1^−^, ${\left(1-g^{2}\right)}^{3}x_{+}\to \frac{27}{4}$. For the lower edge, $x_{-}\to \frac{27}{4}$ can be found by the Taylor expansion of (1 − *g*^2^) or note that (1 − *g*^2^)^3^*x*_+_*x*_−_ = 1. Consider a *x* that is far away from the support edges as *g* → 1, given the above, this means,

$$\begin{array}{c} x\to \infty ,\;x{\left(1-g^{2}\right)}^{3}\to 0. \end{array}$$ (34)

Note that since *x*_+_/*x*_−_ ∼ (1 − *g*^2^)^−3^, there is plenty range of *x* to satisfy the above for strong connections when *g* is close to 1. Under these limits, Eq (5) greatly simplifies as various terms vanish leading to

$$\begin{array}{c} \underset{g\to 1^{-},\;x_{-}\ll x\ll x_{+}}{\text{lim}}p_{C}\left(x\right)/(\frac{\sqrt{3}}{2\pi }x^{-\frac{5}{3}})\to 1. \end{array}$$

This explains the validity of the power-law approximation away from support edges. If we are only interested in the leading-order power-law tail in the critical distribution (i.e., *g* → 1^−^ and *then* *x* → ∞), then there is a simpler alternative derivation that can we also apply to other connectivity models (see Section A.2 in S1 Text).

### 5.4 Comparison with the Marchenko–Pastur distribution

The Marchenko–Pastur distribution is widely used for modeling covariance eigenvalues arising from noise [32, 51, 63]. It is also the limit of the time-sampled spectrum *p*_*g*,*α*_(*x*) (Fig 7 and Section 3.7) at weak connections *g* = 0. The Marchenko–Pastur law has one shape parameter *α*. We focus on the case when the covariance is positive definite which restricts 0 < *α* < 1 (otherwise there is a delta distribution at 0) and the pdf is

$$\begin{array}{c} p_{MP}\left(x\right)=\frac{\sqrt{\left(\alpha_{+}-x\right)\left(x-\alpha_{-}\right)}}{2\pi \alpha x},\;\alpha_{\pm }={\left(1\pm \sqrt{\alpha }\right)}^{2}, \end{array}$$ (35)

The first two moments are 1 and 1 + *α*, from which we know the dimension is 1/(1 + *α*) has a lower limit 1/2. The upper edge *α*_+_ is bounded by 4.

### 5.5 Deterministic connectivity

#### 5.5.1 Ring network with short- and long-range connections

In a ring network, neurons are equally spaced on a circle (can be physical or functional space) and neuron *i* is associated with a location *x*_*i*_ = *i*/*N*, *i* = 0, …, *N* − 1. The connection between two neurons *j* and *i* only depends on the location difference *x*_*i*_ − *x*_*j*_ is therefore translation invariant.

For long-range connections, the connectivity has a shape determined by a fixed smooth periodic function *f*(*x*) on [0, 1),

$$\begin{array}{c} J_{ij}=\frac{1}{N}f\left(x_{i}-x_{j}\right)=\frac{1}{N}f(\frac{i-j}{N}). \end{array}$$ (36)

In the large-network limit, the covariance eigenvalues have an approximate delta distribution at 1 except for a finite number of discretely located larger eigenvalues (Fig 10A). A precise statement of this result is described in Section L.1 in S1 Text. The outlying eigenvalues correspond to the leading Fourier coefficients of *f*(*x*).

For the Nearest-Neighbor (NN) connectivity, only *J*_*i*−1,*i*_ and *J*_*i*+1,*i*_ are non-zero and remain fixed as *N* → ∞.

#### 5.5.2 Multi-dimensional ring network

For a *d*-dimensional ring, the neurons are equally spaced on a *d*-dimensional lattice

$$\begin{array}{c} x_{\vec{i}}=\left(i_{1}/N,i_{2}/N,\ldots ,i_{d}/N\right), \end{array}$$

which is periodic along each coordinate. We focus on the NN connectivity where each neuron is connected to 2*d* neighboring neurons with strength $J_{i_{k}-1,i_{k}}^{k}$ and $J_{i_{k}+1,i_{k}}^{k}$ along direction *k*. We show that the probability density function at both support edges scales as ${\left(\Delta x\right)}^{\frac{d}{2}-1}$ (for comparison, the random network edges scale as ${\left(\Delta x\right)}^{\frac{1}{2}}$). This means for dimension *d* ≥ 2, there is no singularity at the support edges (Fig 10).

To characterize the shape of the covariance spectrum (Fig 10), we further simplify by setting $J_{i_{k}-1,i_{k}}^{k}=J_{i_{k}+1,i_{k}}^{k}=a$ (see also Section L.3 in S1 Text for motivations based on 1D ring) and analytically derived *p*_*C*_(*x*) (Section L.3.2 in S1 Text). For small dimensions *d* ≤ 3, there are distinct “inflection points” within the support. As *d* increases, this non-smooth feature becomes less evident and becomes hard to identify in empirical eigenvalue distributions from a finite-size network (not shown).

### 5.6 Fitting the theoretical spectrum to data

For neural activity data, *C* can be calculated from a large number of time samples of binned spike count *s*_*i*_(*t*) (assuming bin size is Δ*T* large),

$$\begin{array}{c} C_{ij}=\frac{1}{\Delta T}\frac{1}{M-1}\sum_{t=1}^{M}\left(s_{i}\left(t\right)-{\bar{s}}_{i}\right)\left(s_{j}\left(t\right)-{\bar{s}}_{j}\right),\;{\bar{s}}_{i}=\frac{1}{M}\sum_{t=1}^{M}s_{i}\left(t\right). \end{array}$$ (37)

For calcium imaging data, the fluorescence is approximately integrating the spikes over the indicator time constant. So we can still apply Eq (37) by plugging in the fluorescence signal in place of *s*_*i*_(*t*) to calculate the covariance *C* (omit the constant factor Δ*T* which does not affect fitting to the theory, Section 3.8).

We fit the theoretical spectrum to empirical eigenvalues by finding the connectivity parameter *g* that minimizes the error between the *cumulative distribution functions* (cdf) $F\left(x\right)=\int_{-\infty }^{x}p\left(x\right)dx$. This avoids issues such as binning when estimating the probability density function from empirical eigenvalues. We numerically integrate the theoretical pdf (Eq 5) to get its cdf. As seen below, the theoretical cdf only needs to be calculated at the empirical eigenvalues.

Motivated by methods of hypothesis testing on distributions, we measure the *L*^2^ norm cdf error using the Cramer-von Mises statistic

$$\begin{array}{c} D_{CvM}^{2}=\int {\left(F\left(x\right)-F_{n}\left(x\right)\right)}^{2}dF_{n}\left(x\right)=\frac{1}{12n^{2}}+\frac{1}{n}\sum_{i=1}^{n}{\left(F\left(x_{i}\right)-\frac{2i-1}{2n}\right)}^{2} \end{array}$$ (38)

Here *n* is the number of samples and *x*_*i*_ are the *i*-th empirical eigenvalues. Alternatively, the error can also be measured under *L*^∞^ norm based on the Kolmogorov-Smirnov statistic

$$\begin{array}{c} D_{KS}=\underset{x}{\text{sup}}\left|F_{n}\left(x\right)-F\left(x\right)\right|. \end{array}$$ (39)

where *x*_*i*_ are samples. Our code implements both measures.

In Fig 8B–8E, we fit the time-sampled theoretical spectrum with i.i.d. Gaussian connectivity (Section 3.7) to calcium imaging data in larval zebrafish [52]. The theoretical spectrum (once normalized by the mean, see Section 3.8) depends on two parameters *g* and *α*, but the latter is fixed to be *N*/*M* based on the data. Here *N* is the number of neurons in a cluster, and *M* is the number of time frames used in calculating the sample correlation matrix (Eq (37). The calcium fluorescence Δ*F*/*F* traces of each neuron are normalized to z-scores [52], which is consistent with calculating the eigenvalues of the correlation matrix (Section 3.8). *g* is then optimized to minimize the Cramer-von Mises error (Eq 37) between the data. The largest eigenvalue for each cluster is often much larger than the rest and is thus removed before the fitting. For comparison, we fit the same data to the Marchenko–Pastur law (Section 5.4) whose shape depends on the parameter *α*. Here we allow *α* to vary so that both models (random connectivity and MP law) have one parameter to be optimized to fit data.

## Supporting information

S1 Text Supplementary material. Analytical derivations and additional figures.
(PDF)
