# Supplementary material for: The spectrum of covariance matrices of randomly connected recurrent neuronal networks with linear dynamics
Source: PLoS Comput Biol. 2022 Jul 21;18(7):e1010327. doi: 10.1371/journal.pcbi.1010327 (PMC9345493; doi:10.1371/journal.pcbi.1010327)
Supplement: Attachment — Submitted filename: response to reviewers comments.pdf [file pcbi.1010327.s002.pdf]

Responses to reviewers' comments

We sincerely thank the reviewers for their thoughtful and very helpful comments. We have significantly revised the manuscript accordingly. Here is a summary:

- Simulations of nonlinear dynamics and comparison with linear theory (new Sec 4.1 and Fig. 9)
- Discussions of the effect of using calcium signals (new Proposition S11.1 in the supplement)
- Effects of non-normal connectivity in the case of positive kappa_re (new Fig. S8 in the supplement)
- Added plots of dimensionality on the time and space sampled spectra (new insets in Fig. 7), and additional finite-size network realizations and trial-averaged spectrum (new Fig. S1 in the supplement)
- Extended and improved expositions on motivations, results, and derivations in response to reviewers' comments

Below are our detailed responses (in the regular font) along with the original comments (in the bold and italic font). The line, equation, and section numbers are referring to the manuscript file with changes being highlighted (using yellow background or yellow rounded boxes). For the references mentioned below, the numbers are from the main text.

<u>**Reviewer #1:**</u>

The authors calculate the eigenvalue distribution of the covariance matrix of recurrent rate networks linearized around a fixed point driven by additive Gaussian white noise both for i.i.d. Gaussian connectivity and for various non-random connectivities, e.g. symmetric, antisymmetric, low-rank perturbations to i.i.d. Gaussian, ring network and describe the effects of partial temporal or spatial sampling.
The authors fit the eigenvalue distribution to whole-brain Calcium imaging of larval zebrafish and find a better match of the i.i.d. Gaussian connectivity compared to a Marchenko-Pastur distribution.
The contribution of the authors include:
· The analytic PDF of covariance matrix eigenvalues for linear rate networks with Gaussian connectivity.
· A detailed picture of how these distributions are shaped by structural perturbations such as low-rank updates, partial sampling, and partial (anti)symmetry
· A comparison of the eigenvalue distribution obtained from experimental recordings in larval zebrafish to both Marchenko-Pastur distribution and the novel analytically obtained eigenvalue spectrum
· A statement of common characteristics of the covariance matrices and covariance matrix eigenvalue densities, between the analytically solved random linear case and deterministic connectivity cases.

We wholeheartedly recommend this to be published in PLOS CB. The mathematical results are impressive, of high relevance to the audience of PLOS CB, manifold network structures are studied and the results seem mathematically rigorous (especially the Supplement to the extent that we studied it.) However, we feel the utility for the readership of PLOS CB would increase by considering the following major and minor comments:

Major Comments:
· While the mathematical results are very impressive, a detailed discussion of the limitations is largely missing:
- Under what conditions yields the PCA the dimensionality of a data set? (When can we assume the data follows a multivariate Gaussian distribution?). Is that the case for neural data? Why are your findings relevant for neuroscience?

Thank you for the question. Indeed, the dimensionality defined, based on PCA, captures the linear aspects of data distribution and may not accurately reflect the intrinsic dimension of a curved manifold. Nonetheless, PCA is widely used in analyzing neural data as well as theoretical studies (e.g., ref 32,35,43,46,54) and offers useful insights even if the data is not Gaussian distributed. We have accordingly added a qualification of its applicability when introducing PCA and dimension (line 61).

- What are the limitations of a linear response theory? When would it break down?

The general applicability and limitations of the linear response theory has been discussed in the literature, for example, ref 56. To address specifically when the linear response can be used to describe the covariance spectrum, we performed simulations of networks of nonlinear neurons (see details in the new Sec. 4.1, Fig. 9, and Eq. 33). As a summary, when we push the parameters (g and sigma) to make the network more nonlinear, the deviation from the spectrum based on the linear response increases as expected. Interestingly, the simulated spectrum in the nonlinear regimes can still be well described by our theory if we replace g with a reduced effective g_hat, which can be qualitatively understood as due to the average slope of the neuronal nonlinearity. We hope these additional examples of nonlinear dynamics address the question of using linear response theory for deriving the covariance spectrum. A systematic study of the nonlinear case will be carried out in a future work. We also added “linear dynamics” to the title, as suggested below, to make it more clear that our results are based on a linear response framework.

- What is the relationship between ‘linear rate units’ and ‘neurons’. (Often ‘rate units’ are considered as populations of neurons. But how does this fit together with the reference to single neuron motifs (e.g. Song et al. 2005?))

We use the rate neurons as a simplistic model of neuronal dynamics consistent with the approaches the literature (e.g., ref 29). The firing rate can be interpreted as the average spiking

activity of the neuron over a time window. On the other hand, our results on the spectrum would apply to both scales where the units in the network are either neurons or populations of neurons as long as a linear approximation of the dynamics is plausible. There is also evidence of connectivity motifs in biological circuits at coarser levels. For example, see Sporns & Kotter 2004 (new ref 53) and the mesoscale mouse brain example in ref 26. We have added both references when describing motifs (line 230).

- The relevance of the investigation of g approaching 1 from below is not clear. Isn't at g=1 the network dynamics turning unstable and the linear response picture breaking down? Already close to g=1, the fluctuations are getting large, so one might expect that the linear response description is getting increasingly inaccurate because \phi(x)≠x. So while the power-law scaling is an interesting observation, I am not sure it has any relevance for neuroscience (but of course, I'd love to be proven wrong on this).

This is a very good question. We agree that the power-law scaling is partly a theoretical observation that helps to summarize succinctly the differences of the spectrum across different connectivity models, such as, i.i.d. random, symmetric random, and d-dimensional ring. On the other hand, there are also reasons to support the relevance of the power-law phenomenon to neural network dynamics. First, the power law can already provide a good approximation at g=0.7 (Fig.2A). So observing power-law distributed eigenvalues may not require a g very close to the critical value of 1. Second, the large eigenvalues in the covariance when g approaches 1, correspond to large collective fluctuations across the population, which may not warrant a large fluctuation at the neuronal level. Therefore, the linear response may still apply at g close to 1. Indeed, in the nonlinear network simulations (Fig. 9), the spectrum based on the linear theory can well describe the numerical eigenvalues when the effective g_hat>0.7. We have added the above discussions in lines 190, 476.

**** While 'the equations speak for themselves', an intuitive explanation of the observed results and the underlying mechanism is largely missing:***
- Why does g->1 lead to a few dominant eigenvalues in the covariance matrix?

The small number of large eigenvalues is due to the tail being thin in the pdf, as explained around line 204. The thin tail as g->1 can be heuristically understood by considering the case with a normal connectivity J_n with matching eigenvalues to the random J. The large covariance eigenvalues correspond to the eigenvalues \lambda(J^n) that are close to (1,0) on the complex plane, that is, near the right edge of the circular distribution. As g gets closer to 1, the magnitude difference of the covariance eigenvalues become more dramatically dependent on the distance of \lambda(J^n) to (1,0) due to the -2 power exponent in Eq. S74, therefore making the tail of large covariance eigenvalues thinner. We have commented on this in line 205 and added the above discussion in the supplement (after Eq. S74).

- Why do antisymmetry networks have a qualitatively different shape of the distribution of eigenvalues?

Thank you for the question. We have added the following discussion in line 261.
"Since J here is a normal matrix, this qualitative difference from the i.i.d. random connectivity can be understood considering the eigenvalues of J (ref 49), which all lie on the imaginary axis and never approach 1 to cause large eigenvalues in the covariance when increasing g".

- Why do (anti)symmetry networks results in lower (higher) relative dimension D/N? Why does dependence of D(\kappa) change when fixing g_r?

Thank you for the question. As we explained in line 271 (now revised and added with discussions as below), the main effect of symmetry (kappa) on D is due to the change of g_r = g(1+kappa). Intuitively, when kappa increases, g_r is increased and the dimension decreases similar to when g increases in the i.i.d. case. This main effect is removed when we fix g_r. The remaining effect turns out to be D increases with kappa. We do not have an intuitive explanation for this effect beyond the analytical results (Eq. 18).

-The Discussion section seems to add more new results (e.g. reference to ring network and other results in the Supplementary Material) instead of discussing the interpretation of the results and implications for theory and experiments.

The additional results described in the Discussion provide concrete supports to our interpretations. We keep these results there since they are largely tangential to the main results. For implications of our theory, we discussed the robustness of the bulk spectrum to effectively separate signal and noise eigenvalues (line 468), to help distinguish different sources of variability (line 472), and to allow using information from small eigenvalues to estimate effective connectivity (line 490). We have revised these discussions and the new section on nonlinear dynamics (Sec. 4.1) also adds supports to the application of the theoretical spectrum in nonlinear systems.

Minor Comments:
**** Consider changing the title to "The spectrum of covariance matrices of neuronal networks with linear dynamics" or "The covariance spectrum of recurrent neural networks with linear dynamics" or another title that contains the word 'linear' or 'linearly' would make it more clear that here a linear response framework is being used. This also leaves space for future studies that explore nonlinear regimes.***

Thank you for the suggestion. We have added "with linear dynamics" to the title.

**** Figure labels are so small that they are too small. Please increase the label and legend font size to make it readable.***

Thank you for the reminder. We have increased the size of figure labels throughout the manuscript.

**** Make clear that PCA is based on pairwise statistics, so it doesn't require simultaneous recording of all N neurons, it would be enough (assuming stationarity) for all pairs to be recorded one after the other. Therefore, I would like to politely disagree with the notion of 'local' and 'global' features, they might mislead some readers as only two-point interactions are considered here.***

Thank you very much for the comments. Reviewer 2 also raised a related concern. We intended to make a simple distinction that calculating the eigenvalues need the full covariance matrix, whereas, for example, the average correlation can be calculated from a subset of entries from the covariance matrix. We also agree that it is possible to calculate PCA and the covariance matrix from repeated recordings, although this may be inefficient to do experimentally. On the other hand, when considering the effect of limited recording duration for the sample covariance matrix (i.e., the theory in Sec. 3.7), we crucially rely on the assumption that the data are simultaneously recorded (e.g., the C_ij are not independent in this case, but they would be if they were calculated from separate pairwise recordings), which is consistent with majority of experiment scenarios when PCA is done. We have clarified and revised our explanation on this issue accordingly (lines 47 and 55).

**** The comparison of the experimentally obtained covariance spectra of zebrafish data to analytically obtained ones includes assumptions about the temporal correlations of the data (Marchenko-Pastur assumes that samples are independent, i.e. sampled at time frames that are sufficiently far apart such that temporal correlations can be ignored, to what extend is that assumption justified in your data? (It would be helpful to report the frame rate in the caption of the figure 8 and the decay time constant of GCamp6f and the number of time frames used to calculate the empirical covariance matrix. I think your reference 10 has a frame rate of 2.11 per s and GCamp6f has a decay constant of 1796±73ms according to (https://www.ncbi.nlm.nih.gov/pmc/articles/PMC3777791/#SD1) but please check.***

Thank you for the comments. We have added the frame rate in the Fig. 8 caption. This translates to a time bin of 500ms, which makes it reasonable to expect some correlations across bins for the calcium signal. However, as explained below to a related comment, temporal correlations in the calcium may not affect the results for the covariance and eigenvalues, as long as the time bin is long enough to reduce temporal correlations for the spike counts/firing rates. For the spikes, the 500ms window would indeed be sufficient to justify this assumption.

**** The experimentally recorded zebrafish was exposed to visual stimuli and the data was z-scored according to your methods, please comment on how both spatiotemporally structured external input and z-scoring affects your results.***

For the z-score, as we discussed in line 433, this corresponds to studying the eigenvalues of the correlation matrix instead of the covariance. For the theoretical spectrum, the results for the correlation matrix are the same in the large network limit (supplement section S10). We have added a comment on this to line 690.

For the stimulus to the animal, here we only used recordings during the spontaneous window where there is no apparent stimulus but a neural gray background to avoid complications to the neural activity due to visual stimuli (comment added to Fig. 8 caption).

**** Line 126 should read semi-positive instead of positive.***
We have changed to “nonnegative” (line 134).

**** Line 139: Cite earlier papers on participation ratio.***
The ref 43 is an earlier paper cited by ref 32 which uses the participation ratio. Since the concept of participation ratio is intuitive and straightforwardly defined, we find these references sufficient here for showing how the quantity has been used to describe neural activity.

**** Line 160: “Matches pretty well” use precise language.***
Changed to “accurately”

**** Line 167/Figure 1A,B: showing more than one network realization would be more convincing.***
We have added additional network realizations in the new Fig. S1 in the supplement.

**** Figure 1: labels and legend too small***
We have increased the fonts in the figure.

**** Figure 1A,B: consider doing a stair step graph for the empirical histogram across multiple (e.g. 10) network realizations***
We have added plots as suggested in the new supplementary Fig. S1 and referred to it in the main text (line 171).

**** Line 187: c_0 seems not to be introduced in the main text.***
We have modified the equation without introducing the c0 to avoid confusion (line 199).

**** When you refer to Supplement, it would help if you refer to the section of the supplement because it is 60 pages long so things would be easier to find.***
We have added section/figure/equation numbers when referencing the supplement.

**** Figure 5: inset too small, axes not legible***
We have increased the labels on the inset.

**** Line 296: only K<>1 for the Gaussian assumption.***
Sorry, but we are not very clear about this comment.

**** It would be nice to plot both D(f) and D(\alpha) (equation 23) next to figure 7AB so the effect of sparse time sampling becomes even visually more obvious.***
Thank you for the suggestion. We have added plots of the dimension as insets in Fig.7 AB.

**** A closer look at the methods section (5.6) indicates that the authors incorporated temporal sampling corrections in their calculation of the theoretical spectral CDFs used to fit the neural data. However, it is not clear in the main text (3.8) that they did this.***
Actually, we stated this in the main text (line 452) as well as in Fig. 8 caption (when describing panel B). We have added in the caption "time-sampled" also for panel C-E to make it clear.

**** In section 3.8, where the authors fit distributions to data, a discussion of the data is missing. Would you expect the same PCA distribution for spikes instead of calcium? What is the effect of the Calcium response? It would be good to comment on the fact that the eigenvalues of a covariance matrix change after a change of variable.***

Thank you for the question. Under the assumptions that: (i) calcium is linearly related to the spikes by convolving a temporal kernel (ii) the time bins are long enough for the spikes such that there is no correlation for the spikes (not the calcium) cross bins, one can show that the covariance matrix calculated from calcium is just a constant multiplying the covariance calculated from the spikes. So changing the variables in this case would not affect the eigenvalue distributions. We have added a comment on this in the main text (line 453) and a section on the derivation in the supplement (Proposition S11.1).

**** In figure 8, important details are missing: How many data points are used to estimate the covariance matrix? Why don't you fit the model including symmetry/antisymmetry? What D/N do you numerically estimate for the different clusters? Do you have full access to all neurons Calcium activity? If not, do you apply your spatial sparsity model?***

Thank you for the questions. We have added the number of data points (600 time frames) to Fig. 8 caption and the estimated dimensionality for each cluster (in the legends for the empirical eigenvalues). The experiment (ref 10) records roughly 80% of all neurons but the coverage differs from region to region. We agree that ideally a spatially subsampled model could be considered, as well as the model with the symmetry parameter kappa. Due to the nature of this preliminary-data application being a proof-of-concept, we did not consider these models here to keep the theory as simple as possible, and also to have the same number of

tuning parameters (one) to compare fairly with the MP law. A systematic analysis of this zebrafish data and other experimental datasets using the theory is left for future work.

***** Instead of motifs-> second-order motifs. (there are also higher order motifs)**
We have changed to "second-order motifs" in the abstract and lines 73, 233, 465.

***** Please go carefully over all references to correct typos and misspelled names.**
Thank you for the reminder. We have gone through the references and corrected them.

Stylistic:
***** "iid" -> "i.i.d."**
We have changed this throughout.

***** Highly complex->complex**
We have changed as suggested.

***** "Frequency spectrum in Fourier transform"-> "Frequency spectrum obtained via Fourier transform"**
We have changed as suggested.

***** "co-fluctuations" doesn't to my knowledge exist, maybe use covariation instead?**
We changed in line 87 to "neuronal variability" and to "covariation" in line 103.

***** Instead of "the network's Principal Component Analysis, we would propose the network dynamics Principal Component Analysis.**
We have revised as suggested.

***** Line 237: For denoting the imaginary i, I would not use a double-struck i.**
We have changed to regular "i" throughout.

Typos:
***** Whitespace line 71 after dynamics.**
***** Excitatory-Inhibitory-> excitatory-inhibitory**
***** Line 559: "random matrix" -> "random matrices"**
***** In abstract: "The theoretical results are compared with those from finite-size networks and and the effects" should read "The theoretical results are compared with those from finite-size networks and the effects".**
***** "the rank plot with exponent"**
***** "supported by a NIH grant" -> "supported by an NIH"**
***** "which correspond to overabundance of certain subgraphs" -> "which correspond to an overabundance of certain subgraphs"**
***** "as g approach the critical"-> "as g approaches the critical"**
***** "In large-network limit"-> "In the large-network limit"**

**** “the error can also be measure under” -> “the error can also be measured under”***
**** “Storing Infinite Numbers of Patterns in a Spin-Galss Model of Neural Networks”-> “Storing Infinite Numbers of Patterns in a Spin-Glass Model of Neural Networks”***
**** “Multiplciation of certain noncommuting random variables”. -> “Multiplication of certain noncommuting random variables”.***

Thank you so much for the thorough reading and corrections. We have address these accordingly.

Reviewer #2:

Summary:

In this manuscript, Hu and Sompolinsky derive analytical expressions for the eigenvalue spectrum of covariance matrices and the related dimensionality measure (participation ratio) for random networks (iid weights / motifs / excitatory-inhibitory, and also some structured networks for comparison). Their theory is based on linear(ized) dynamics, where there is a simple relation between covariances and connections. Yet, for the general case of non-normal connectivity matrices, the derivation of the covariance spectrum from connectivity parameters is involved. Their theoretical derivations, that for better readability are mostly presented in the extensive supplement, are based on the seminal work by Sommers, Crisanti, Sompolinsky and Stein in 1988.

Given the large interest in dimensionality of neural activity in the computational neuroscience community over the recent years, this work is highly relevant and timely and an important contribution to the understanding of the relation between structure and dynamics in neural networks. The presentation is mostly clear, the theoretical derivations are solid and the authors also show an application to experimental data (whole-brain calcium imaging data from larval zebrafish). We therefore believe this paper should eventually be published in PLOS CB. There are, however, a few major and minor issues (see below) that need to be resolved in a revised version before the manuscript is ready for publication.

Major points:

Effect of other motifs:

The authors study in depth the effect of reciprocal connections / symmetry of connections on the covariance spectrum. They also discuss the other motifs (divergent, convergent, chain), but state that they do not "affect the bulk spectrum of C" (main text line 220). In the supplement below Eq. S26 the authors, however, state that also divergent, convergent and chain motifs affect the bulk spectrum indirectly by channeling their effect through changing the normalized

reciprocal motifs. Can the authors clarify this and elaborate in more detail in the main text, which motifs affect the bulk spectrum in which way? Also the critical coupling strength / stability should depend on all motifs (see Eq. S26), so one would expect an influence of all motifs on the bulk covariance spectrum.

Thank you for the comments. Because of the way the motif magnitude parameters (\hat{\kappa}'s) are defined involve being normalized by the variance of connection weights, we need the values of diverging, converging, and chain motifs to calculate the parameters g and kappa for the reciprocal-only component \tilde{J}, and then calculate the covariance spectrum using the theory in Sec. 3.3.2, which depends only on g and kappa (of \tilde{J}). In this sense, these three motifs affect the bulk covariance spectrum indirectly. As an example, consider the following two scenarios. First, one can add diverging motifs to a reciprocal-only \tilde{J} by adding a low-rank matrix eb^T. This addition changes multiple parameters together, i.e., g^2(J) and \hat{\kappa}_{div}(J), such that the g^2(\tilde{J}) and \hat{\kappa}_{re} (\tilde{J}) for \tilde{J} remain the same (when calculated using Eq. S26), and so is the bulk spectrum. Now, assume that there is another modification of the connectivity matrix such that only hat{\kappa}_{div}(J) is increased while all other parameters of J stay the same, then the bulk spectrum will change, because g^2(\tilde{J}) and \hat{\kappa}_{re} (\tilde{J}) will change according to Eq. S26.

We have revised along these lines in the main text (line 234) and the supplement (line 213) to clarify this issue.

Clarification of limitations:

In Section 3.5 the authors should stress more that their theoretical predictions are limited to networks where all connections have identical variance. Their choice of E-I network is a special case that is explicitly constructed such that this requirement holds. In typical E-I networks the variance is not the same due to different population sizes, connection probabilities, and synaptic strengths (that are not precisely tuned to achieve the same variance of E and I connections).

We completely agree. We have added an emphasis on this requirement in the main text (line 333): "Importantly, the choice of connection probabilities and weights ensures that var(J_ij) is w_0^2/N (to the leading order for N>> 1) regardless of the cell type of neuron i,j This allows us to define the effective synaptic gain as g^2=w_0^2 for all neurons."

Relation to Sommers et al. 1988 (S1988):

***--------------------------------------

In supplement S1 the authors show how to derive the probability density p_c for the covariance eigenvalues from the potential Phi that has been computed in S1988. What did not become clear to us is how the derivation deviates from the calculations in S1988. Because S1988 calculated the probability density p_J of the connectivity spectrum rather than p_c. Both calculations seem to rely on the precision matrix, but the detailed differences in calculations did not become clear from reading the supplement. Can the authors please provide more details in which steps of the calculations results from S1988 are exactly used and in which steps results needed to be adapted to treat the covariance spectrum rather than the connectivity spectrum?

Thank you for the comment. The key difference is that in S1988, the regularizing parameter epsilon is taken to 0 after Eq. S6 (Eq.19 in S1988), which is sufficient to derive the spectrum of J because lim_{epsilon->0+} Phi = log(det(P)) =2 log(|det(eta*I-J)|). In contrast, here we need to make use of general values of epsilon (and beyond positive real values), in Phi=log(det(epsilon*I+P)) to find the spectrum of the precision matrix P. We have accordingly elaborated on this point in the supplement (lines 48 and 56).

Relation to Stringer, Pachitariu et al. Nature 2019 (SP2019)
--

In line 188, the authors present their results as an alternative mechanism for the experimentally observed power laws of covariance spectra in SP2019. The authors, however, study the covariance structure of spontaneous network dynamics while SP2019 studies stimulus-evoked activity (responses in V1 due to visual stimuli). Would one thus not expect differences in covariance spectra? Can the authors briefly elaborate on this?

We totally agree with the comment. To relate to SP2019, we need to motivate the covariance matrix Eq. 2 from a different network model where neurons are linearly driven by whitened stimuli and reach their steady state during the recording (i.e., the model in line 601 in Methods). We have revised and noted this point in the main text (lines 117, 202, 607):
“These models provide additional motivations for the covariance (Eq. 2) which may allow interpreting our results in experiments where the neural activity is driven by stimuli SP2019.”
“This perspective is needed to use our results to interpret experimental data where the neural activity is largely driven by stimuli for example SP2019.”

Relation to Dahmen, Recanatesi et al. biorxiv 2020 (DR2020):

In line 208 the authors state that the dimensionality result Eq. 10 can also be derived based on results of their reference [11]. This has already been done in DR2020 (https://www.biorxiv.org/content/10.1101/2020.11.02.365072v1.full.pdf). It would be helpful to explain the relation of the current work to these parallel developments. This seems particularly important given the high similarities between figures of the current manuscript and figures in DR2020, in particular
-Fig 1D <-> DR2020:Fig 2B
-Fig 4B <-> DR2020:Fig 4B
The approximation in l.257 for D/N as a function of g_r corresponds to the approximate result in DR2020.
Also in the supplement "First two moments as a corollary of results in [3]" it would be helpful of the authors cited DR2020 to clarify the close relation of these parallel works.

Thank you very much for pointing us to this reference DR2020, which is indeed very relevant. We were not aware of this independent work when developing our results and writing the manuscript. We have added a reference to DR2020 and a brief note in the Discussion (lines 466 and 223) on what results are potentially in common in both studies and what results in the current work are not in DR2020: “Some of these dimensionality results are also derived in a recent parallel work DR2020, whereas the shape of the covariance spectrum was not studied in DR2020.”

Motivation of the manuscript:
--

To motivate their work, the authors dinstinguish in the first paragraph of the introduction between local and global features of dynamics. They cite a few works falling in the local feature category and argue that their work targets the global scale.

We, however, disagree with the authors' categorization. While their refs [55,48] really discuss local features, refs [27,11,18] study correlations (mean and variance across populations). The latter emerge from collective network effects and should thereby be attributed to the global feature category. In fact, since the dimensionality and covariance spectrum are based on the very same covariances, the authors' work falls in the same category as [27,11,18].

Thank you for the comment and we agree with these statements. Please let us clarify the "local" vs "global" distinction we tried to make. As we replied to Reviewer 1 who raised a similar issue, we intended to make a simple distinction that the calculation of the eigenvalues needs the full covariance matrix, whereas, for example, the average correlation can be calculated from a subset of entries of the covariance matrix. This difference means that calculating the covariance spectrum requires simultaneously recorded neural activity (thus "global" or "joint") vs repeated recordings of a small number of neurons each time ("local" or "marginal"). In principle, the covariance matrix can also be calculated from repeated recordings, but this may be inefficient to do experimentally. Furthermore, when considering the effect of limited recording durations on the sample covariance matrix (i.e., the theory in Sec. 3.7), our analysis crucially relies on the assumption that the data are simultaneously recorded (e.g., the C_ij are not independent in this case, but they would be if were calculated from repeated pairwise recordings), which is consistent with majority of experiment scenario when PCA or the covariance matrix is calculated. We have clarified and revised our explanation on this issue in the main text accordingly (lines 47 and 55).

Minor points:

Power law exponent:

Why is the power law shape of the covariance spectrum not continuous in the parameter kappa? I.e. why is there a single power law exponent for -1kappa=-1? Are these results an artefact of the calculations that are different for kappa \in {-1,1} and -1numerical comparisons for the power law exponents for various kappa?
The authors should extend their discussion on these points.

The qualitative difference for kappa = -1 or 1 is not a numerical artifact and we derive the asymptotic power laws under all kappa analytically (Sec. S5.1 in the supplement). Intuitively, the special case of kappa = -1 or 1 is consistent with that the spectrum of the connectivity J being distributed in 1D rather than in an ellipse for -1<kappa<1. We have added a discussion on this in line 281:
"In comparison, the kappa=\pm 1 are singular cases in Eq.17 and have a different limiting power-law behavior (an exponent -4/7 and no long tail). This is intuitively consistent with the spectrum of J which becomes confined on a line for kappa=\pm 1 rather than in an ellipses for -1<\kappa<1 (Sommers1988)."

Wrong cross-references in the supplement:

Please check again the cross-references in the supplement. Some of them textually refer to Figures in the main text, but the hyperlink takes us to figures in the supplement, e.g. in l.474: Ref to Fig 6 (in the main text) wrongly links to Fig. S6 in the supplement.

This is due to a Latex issue. We have resolved it and the external reference hyperlinks now will open and point to the other file.

Figures:

Fig 1: Colors for theory and simulation not consistent across panels
We have changed the color to be consistent.

Fig 4A, Fig 5C&D, Fig 6A&B: legend labels for lines not consistent: decide for "theory" or "Gaussian theory"
We have changed the labels to “theory”.

Fig. 6: Better explain the correction with the modified connection weight.
We have added an explanation in Fig. 6 legend: the modification is to make var(J_{ij})=w_0^2/N hold exactly for finite N.

Fig. S6: y-axis labels are cut off
We have corrected this.

Strong non-normal effects:

Sections S5.2 and S5.3: I am not sure which point the authors want to make here. The bimodality of the spectrum is an interesting observation, but the comparison to the covariance spectrum of the matching normal connectivity shows that the bimodality is not an effect of the non-normality. In contrast the bimodality is more severe for the matched normal. So the non-normality seems to suppress the bimodality. If this is the main message then the authors should write this more clearly in the supplement and also in Sec 3.3.2 of the main text. How do the covariance spectra and matched normal spectra compare for kappa_re>0?

Thank you for the comment and suggestion. We agree and have added a clear statement of "suppression of a bimodality via non-normal J" in both the main text (line 300) and supplement (line 447).

For kappa>0, the non-normal effect of the connectivity is weaker. We have added a figure with examples and the following discussion in the supplement (Fig. S8, line 459):
"In comparison, the non-normal effect of the connectivity is weaker for kappa_re>0. This is illustrated by the examples in Fig. S8, where, as in Fig.S7 B, we compare the actual covariance spectrum with the one based on a matching normal connectivity for kappa_re >0. The matching normal spectrum is qualitatively similar, and the similarity increases with kappa_re which is consistent with kappa_re =1 corresponding to a normal J. The similarity also increases with g_r for small eigenvalues, but not for the large eigenvalues (note the edge of the spectrum support in Fig. S8 marked by solid dots, and see also the discussion around Eq. S79)."

Time and space sampling:

We found supplemental sections S8 and S9 hard to follow, especially for readers that are not familiar with free probability theory. We are aware that the authors cannot go into all details on this issue in this manuscript, but it would be helpful for the average reader of PLOS CB if the authors could provide even more intuition on the various steps of the calculations and possibly single out a good references for an overview article that introduces the concept of free prbability theory.

Thank you for the comment. For a quick overview of free probability, we recommend a review chapter that is also available online (newly added reference 52). We have added this reference (line 392) and additional explanations in S8 and S9 (lines 554, 562, 573, 578, 596, 602, 653, 680) in the supplement.

References:
----

l.272: We think it would be suitable to also cite Schuessler et al. NeurIPS 2020, which showed that training random networks creates low-rank connectivity components.

Thank you for the suggestion. We have added the reference (line 307):
"…and by training neural networks by gradient decent [Schuessler2020]."

More detailed minor points (Supplement):

Eq.S3: indicate that -z is the argument corresponding to epsilon in Eq.S2, i.e. d Phi/d epsilon(-z,\eta)

We have modified as suggested.

In Eq. S2 epsilon shall be larger than zero in, but z can take on all values in C excluding the real line. Could you please explain this a bit more in detail?

We have revised the explanation on this in line 61:

"Note that although the definition of Φ (Eq. (S2)) restricts $z = -\varepsilon < 0$, the Stieltjes transform is analytic in z outside of the support of p_η(x). Since Eq. (S7) only involves the Stieltjes transform, its validity can be extended from $z < 0$ to any complex z not on the support of p_η(x) (which is part of the positive real axis). "

l.165-166: Please define F_C also here in supplement.

We have added the definition in line 174.

Eq.S26: It it worth noting that \hat{kappa}_*(J) is normalized by the variance of J and \hat{kappa}_*(\tilde{J}) is normalized by the variance of \tilde{J}

Thank you for the suggestion, we have added that in line 215:

"note that κˆre(tilde{J}) is normalized by var(tilde{J}) whereas k_re(J) is normalized by var(J)."

Eq.S27: It is not clear where Sylvester's identity has been applied. What is A and B in eq. S27?

We have added an explanation in line 228:

"Here we used Sylvester's identity $\det(I - AB) = \det(I - BA)$ in the last equality to exchange the order of multiplying u and $u^T (zI - C)^{-1}$. "

l.340-341: z=eta? Please remind the reader here on the relation between p_C and p_eta (shown in l.81).

Yes and we have corrected that and restated the p_C and p_eta relation there (line 356).

Eq.S66: How to get from mu_1 to mu_1(C)?

There was a typo in line 408, where the M(z) and mu_1, mu_2 should correspond to P^{-1}=g^2 C, not P as was in the previous version. So mu_1(C) simply is mu_1/g^2 and substitute the parameter notations. We have corrected this.

l.426 and 436: Ref to Fig 3B correct?
We have checked and these references are correct. Fig. 3B is the antisymmetric connectivity curves corresponding to kappa=-1.

Eq.S75: dx missing on the rhs?
We have corrected this by removing dx from the rhs (now S76).

l.513-514: what are overlines over omega and C referring to?
It is the complex conjugate. We have added the definition in line 539.

Typos (Main text):
----

l.22: and and -> and
l.125: the -> The
l.140: two moments -> two moments of
l.272: .[33,44]. -> [33,44].
l.528: broken sentence
l.536: weights) -> weights
l.559: random matrix -> random matrix theory
l.619: pdf -> cdf
l.623: measure -> measured

Typos (Supplement):
----

l.121: E(\lambda)^n -> E(\lambda^n)
l.129: the its -> its
l.208: comma missing in between inline equations
l.354 and in following sections: p_P(x)->p_eta(x)
l.476: w0 ->w_0
l.523: C\omega)->C(\omega)
l.533-534: g missing in equation?

Thank you so much for the corrections. We have addressed them accordingly. For the question about l.533-534, J contains g and J0 has 1/N var as in other parts and Eq. S1. So we removed the g in Eq. S83 and the ensuing equation.

Reviewer #3:

The manuscript analyzed theoretically the eigenvalue distribution of covariance matrix of the spontaneous neural activities in the randomly connected recurrent neuronal networks, and found the distribution has a finitely supported smooth bulk spectrum and exhibits an approximated power-law tail for coupling matrices near the critical edge. The simulation results consist with the theoretical prediction. I believe the mathematical derivations are rigorous and the results are solid.

However, I have some major concerns.
1. To analyze the eigenvalue distribution of covariance matrix, the authors made some important simplifications, for example, both the interactions between neurons and local dynamics of single neurons are linear. However, the local dynamics and interactions of our neural systems are all nonlinear, and lots of computing advantages are emerged based on these nonlinear characteristics. So could the authors provide some more simulation results to support that the main results can be extended to the nonlinear neural networks.

Indeed, our results are based on the simplifying assumption of linear dynamics, and we have further emphasized this by adding "linear dynamics" to the title. We believe that a linear theory of the covariance spectrum is a useful first step with many interesting results and is in line with literature (e.g., ref 44). We totally agree that it is important to study network models with strong nonlinear dynamics, but this is best left for future work. As a proof-of-concept, we performed simulations of networks of nonlinear neurons (see details in the new Sec. 4.1, Fig. 9, and Eq. 33). To summarize the results, as we push the parameters (g and sigma) to make the network more nonlinear, the deviation from the spectrum based on linear response increases as expected. Interestingly, the simulated spectrum in the nonlinear regimes can still be well described by our theory if we replace g with a reduced effective g_hat, which can be qualitatively understood as due to the average slope of the neuronal nonlinearity. In sum, these additional examples provide support for the applicability of our linear theory to nonlinear dynamics

2. I am not clear what the eigenvalue distributions could give some new insights to the neural information processing mechanism? For example, the first few eigenvalues and eigenvectors of the covariance matrix can reflect the main computing characteristic or features of neural activities, so could the authors address the new computing advantages or functional meaning reflected by the eigenvalue distributions to the dynamics of neural activities?

Thank you for the question. First, as discussed in the Introduction (lines 59, 63), the dimensionality of neural activity has recently received a lot of interest including both experimental observations and theoretical studies (ref 32,35,44,48,52) illustrating its roles including representing stimuli (ref 13) and generating motor outputs (ref 16). A widely used measure of dimensionality (Eq. 4) is calculated using all the covariance eigenvalues, or equivalently from the eigenvalue distribution.

Second, the shape of the distribution of all eigenvalues, including the large and small ones, reflect the properties of the recurrent interactions, such as the effective connection strength (g, Fig. 1C) and motif frequency (kappa, Fig. 4D). We have added a comment on this in line 490:
"By fitting experimental data to the theoretical spectrum, eigenvalues of all sizes may be used to estimate this information about the functional connectivity (Fig. 8). "

Third, partially related to the question, understanding the distribution of smaller eigenvalues may help identify the large eigenvalues in experimental data (line 468):
"Knowing the exact shape and support of the bulk spectrum can facilitate separating outlying eigenvalues corresponding to low dimensional structure (coming from other unmodeled effects such as external input) from variability due to noise \cite{Peyrache2010} (see an example in Fig. S13 in the supplement)."
This application is also supported by the theoretical results in Sec. 3.4 on the robustness of the covariance spectrum to low-rank perturbations (potentially the large eigenvalues).

3. The manuscript analyzed the covariance matrix of spontaneous activity, however, the neural systems usually show persistent activities when our brain performs some cognitive functions. Could the results extend to the study of persistent activities?

The basic assumption of our model (Eq. 1) is that the neural activity is stationary and fluctuate around a fixed point (line 80). Experimentally this can be related to persistent or spontaneous activity when there is no structured spatial-temporal stimuli. We have added a comment on this in line 81:
"The dynamics considered here is simple where the activity fluctuations around the steady-state are described by a linear response \cite{Lindner2005a,Trousdale:2012}, which experimentally is related to spontaneous or persistent neural activity in absence of structured spatial-temporal stimuli."

Our framework, however, is not applicable to situations when the persistent neural activity is not stationary in time, i.e., being transient. This is an interesting and important direction that is left for future work. We have added a comment on this in the Discussion (line 515):
"Future work could also consider cases with transient activity which are common in nonlinear systems…"
