# Supplementary material for: The spectrum of covariance matrices of randomly connected recurrent neuronal networks with linear dynamics
Source: PLoS Comput Biol. 2022 Jul 21;18(7):e1010327. doi: 10.1371/journal.pcbi.1010327 (PMC9345493; doi:10.1371/journal.pcbi.1010327)
Supplement: Attachment — Submitted filename: response to reviewers comments_2.pdf [file pcbi.1010327.s003.pdf]

Responses to reviewers' comments

We thank the reviewers for confirming that our revision has resolved most of their concerns and for their additional comments and suggestions. We have revised the manuscript accordingly. In particular,

- We have added in the supplementary materials a figure (S7) and discussions on the power-law approximation under different kappa's.

Below are the reviewers' original comments (in the bold and italic font) and our detailed responses (in the regular font). The line, equation, and section numbers refer to the latest manuscript file with highlighted changes (using yellow background or rounded yellow boxes). For the references mentioned below, the numbers are from the main text (re-ordered as we switched to using the PLOS citation style file).

Reviewer #1:

General evaluation:
I thank the authors for addressing all major comments and questions. The manuscript is now much more clear and understandable. The additional figures greatly improve the presentation. I agree with the change of the title and consider the finding sufficient for publication in this journal.

Thank you very much for the confirmation and for reviewing our submission.

There are just one minor issue remaining:

Minor comment:
Line 264: Instead of "Since J here is a normal matrix, this qualitative difference from the i.i.d. random connectivity can be understood considering the eigenvalues of J [49], which all lie on the imaginary axis and never approach 1 to cause large eigenvalues in the covariance when increasing g."
I would suggest writing instead for enhanced clarity: "Since J here is a normal matrix, this qualitative difference from the i.i.d. random connectivity can be understood considering the eigenvalues of J[49]. The eigenvalues of J all lie on the imaginary axis and therefore never approach 1. Thus, the eigenvalues of the corresponding covariance matrix don't develop a long tail."

Just for clarification: Our previous comment about line 296 was erroneous.

Thank you for the suggestion. We have modified the sentence as suggested:
Line 264: "Since J here is a normal matrix, this qualitative difference from the i.i.d. random connectivity can be understood considering the eigenvalues of J [31]. The eigenvalues of J all lie on the imaginary axis and therefore never approach 1. Thus, the eigenvalues of the covariance matrix do not develop a long tail when increasing g."

__Reviewer #2:__

We thank the authors for addressing most of our comments in detail in the revision. The presentation of results has much improved. There are, however, a few points that are still unclear and need to be clarified before the manuscript is ready for publication in PLOS CB.

Power law approximation:

We apologize that parts of our previous question on the power-law approximation seem to have been cut during the submission of the comments. The missing part concerned the non-continuousness of the power law exponent with kappa. We find unintuitive that there seems to be an abrupt change in the distribution of bulk eigenvalues from e.g. kappa=1- to kappa=1 (likewise for the other extreme case kappa=-1). Because the approximated theory predicts different power law exponents. Can the authors show simulations of p_c in logarithmic yscale for readers to see what happens to the power law for fixed g_r and varying kappa over the whole range including the edge cases kappa=-1 and kappa=1 (Similar to Fig 4D but with log scale on y axis and including cases closer and including kappa=+-1)? We understand that the topology of connectivity eigenvalues changes from 2D to 1D at the limiting kappas, but this happens in a smooth manner, so we would have thought a smooth change in the covariance spectra.

Thank you for the comment and suggestion. To elaborate on this matter, we have added a figure as suggested (Fig. S7, copied below) and discussions like below to the supplement (line 440 to 458), which we referred to in the main text (line 285).

The key to understanding the discontinuity is to note that the power-law approximation involves two limits: first taking gr to 1 and then taking x to infinity (also in line 650 of the main text). Near kappa=1, for a fixed gr and any **fixed x**, the spectrum indeed changes continuously with kappa (Fig. S7A). However, the limiting order of p(x) as x goes to infinity is not continuous with respect to kappa at 1. To help illustrate the situation, consider a toy example of p(x) = x^{-a} + (1-kappa) x^{-b}, a>b>0. This function changes continuously with kappa for any fixed x, while the limit of x goes to infinity has a power law exponent of b for kappa<1 and an exponent of a for kappa=1.

In the numerical plot Fig. S7A, please note that the slope difference of -5/3 and -7/4 is small (-1.67 vs. -1.75, the two-colored dashed lines). Therefore, we used a gr close to 1 to better visualize the difference between the two power laws. With a fixed gr<1, the power-law approximation applies when x is far away from the (right) edge of the distribution. The pdf curves with different kappa’s depart from the power law (blue dashed line) for smaller x when

kappa is closer to 1. For kappa=1, the pdf does not approach the power-law line with exponent -5/3 (blue) for large x, but instead approaches the -7/4 power law (black dashed line).

Near kappa = -1, for a fixed gr, the spectrum maintains an approximate power law with exponent -5/3 but shifts downwards in the log-log plot (i.e., reduces in magnitude, Fig. S7B). This is intuitively consistent with the constant factor of (1+kappa) in Eq. 17. In the limit of kappa = -1, the magnitude of the power-law approximation vanishes. Note that for kappa = -1, gr = g(1+kappa) is always 0, and hence it is not possible to include a kappa = -1 curve in Fig. S7B when fixed at a positive gr.

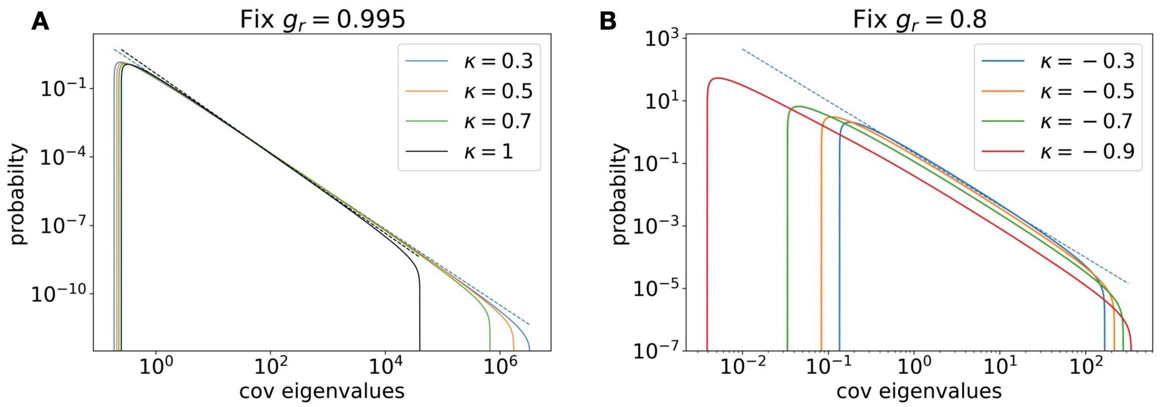

Figure S7: **Power-law approximations for different κ. A**. The pdf of the covariance spectrum in the log-log scale under different κ near 1, while fixing $g_r = g/g_c = g(1+\kappa)$. The blue dashed line is the power law Eq. (S74) with $\kappa = 0.3$. The black dashed line is the power law Eq. (S55). Note that the slope difference of the two power laws is small (-1.67 vs. -1.75). Therefore, we use a g_r close to 1 to better visualize their difference. **B**. Similar as A. but for κ near -1. The blue dashed line is the power law Eq. (S74) with $\kappa = -0.3$.

Nonlinear network:

The analysis of the nonlinear network is a nice and convincing addition to the manuscript. We, however, do find the authors' description in lines 514ff potentially misleading. There the authors state:

"Note that even for g > 1, the dynamics examined here is largely driven by the noise rather than the intrinsic chaotic dynamics studied in [50]."

It is not surprising that the linear approximation of the dynamics and spectrum work well even for the case g>1 studied in Fig. 9. The dynamics of all networks shown in Fig. 9 are not chaotic. In fact, the dynamics are even linearly stable. As shown in Molgedey et al. PRL 1992 for discrete-time networks and in Schuecker et al. PRX 2018 for continuous dynamics (same model as studied in this manuscript) the

transition to linear instability and the transition to chaos are both shifted to larger values of g in the presence of external input (cf. e.g. Fig. 3 in Schuecker et al.). Maybe the authors had this in mind, but they should make the statement more precise to not suggest that the approximation works well for networks with chaotic dynamics.

We fully agree with the reviewer's comment that the cases we studied in Fig. 9 are not chaotic dynamics, which is also what our statement in line 514 intended to mean (see also the sentence in line 516, we mentioned that studying the covariance spectrum in chaotic dynamics is a direction left for future work). We have modified the sentence to clarify this (see below). We also thank the reviewer for pointing to us the highly relevant paper by Schuecker et al. We have added a citation to it (ref 56) in line 504 and 515.

Line 514: "Note that, unlike the noiseless model in [2], the cases studied here are not chaotic even with g>1. As shown in [56], the presence of external noise shifts the transition to chaotic dynamics from g=1 to larger values."

Minor points:

The xlabel in Fig 1D is missing.

Typos:
------
l479: a concise theoretical characterizations -> concise theoretical characterizations
l619: firing rates r_i(t) is binned -> firing rates r_i(t) are binned
Supp l.215f: This does not contradicting -> This does not contradict
Supp l.217: one introduce -> one introduces

Thank you for the careful reading and the corrections. We have addressed them accordingly.

Reviewer #3:

I thank the authors for a thorough revision and comprehensively answering all of my questions. I have no further concerns.

Thank you very much for the confirmation and for reviewing our submission.
