# Supplementary material for: The spectrum of covariance matrices of randomly connected recurrent neuronal networks with linear dynamics
Source: PLoS Comput Biol. 2022 Jul 21;18(7):e1010327. doi: 10.1371/journal.pcbi.1010327 (PMC9345493; doi:10.1371/journal.pcbi.1010327)
Supplement: Peer review history

# Peer review: Decision Letter 0

**DOI:** 10.1371/journal.pcbi.1010327.r001

**Authors:** Thomas Serre, Tianming Yang

16 Feb 2022

Dear Dr. Hu,

Thank you very much for submitting your manuscript "The spectrum of covariance matrices of randomly connected recurrent neuronal networks" for consideration at PLOS Computational Biology.

As with all papers reviewed by the journal, your manuscript was reviewed by members of the editorial board and by several independent reviewers. In light of the reviews (below this email), we would like to invite the resubmission of a significantly-revised version that takes into account the reviewers' comments.

We cannot make any decision about publication until we have seen the revised manuscript and your response to the reviewers' comments. Your revised manuscript is also likely to be sent to reviewers for further evaluation.

When you are ready to resubmit, please upload the following:

[1] A letter containing a detailed list of your responses to the review comments and a description of the changes you have made in the manuscript. Please note while forming your response, if your article is accepted, you may have the opportunity to make the peer review history publicly available. The record will include editor decision letters (with reviews) and your responses to reviewer comments. If eligible, we will contact you to opt in or out.

[2] Two versions of the revised manuscript: one with either highlights or tracked changes denoting where the text has been changed; the other a clean version (uploaded as the manuscript file).

Important additional instructions are given below your reviewer comments.

Please prepare and submit your revised manuscript within 60 days. If you anticipate any delay, please let us know the expected resubmission date by replying to this email. Please note that revised manuscripts received after the 60-day due date may require evaluation and peer review similar to newly submitted manuscripts.

Thank you again for your submission. We hope that our editorial process has been constructive so far, and we welcome your feedback at any time. Please don't hesitate to contact us if you have any questions or comments.

Sincerely,

Tianming Yang

Associate Editor

PLOS Computational Biology

Thomas Serre

Deputy Editor

PLOS Computational Biology

***********************

Reviewer's Responses to Questions

**Comments to the Authors:**

**Please note here if the review is uploaded as an attachment.**

Reviewer #1: The authors calculate the eigenvalue distribution of the covariance matrix of recurrent rate networks linearized around a fixed point driven by additive Gaussian white noise both for i.i.d. Gaussian connectivity and for various non-random connectivities, e.g. symmetric, antisymmetric, low-rank perturbations to i.i.d. Gaussian, ring network and describe the effects of partial temporal or spatial sampling.

The authors fit the eigenvalue distribution to whole-brain Calcium imaging of larval zebrafish and find a better match of the i.i.d. Gaussian connectivity compared to a Marchenko-Pastur distribution.

The contribution of the authors include:

· The analytic PDF of covariance matrix eigenvalues for linear rate networks with Gaussian connectivity.

· A detailed picture of how these distributions are shaped by structural perturbations such as low-rank updates, partial sampling, and partial (anti)symmetry

· A comparison of the eigenvalue distribution obtained from experimental recordings in larval zebrafish to both Marchenko-Pastur distribution and the novel analytically obtained eigenvalue spectrum

· A statement of common characteristics of the covariance matrices and covariance matrix eigenvalue densities, between the analytically solved random linear case and deterministic connectivity cases.

We wholeheartedly recommend this to be published in PLOS CB. The mathematical results are impressive, of high relevance to the audience of PLOS CB, manifold network structures are studied and the results seem mathematically rigorous (especially the Supplement to the extent that we studied it.) However, we feel the utility for the readership of PLOS CB would increase by considering the following major and minor comments:

Major Comments:

· While the mathematical results are very impressive, a detailed discussion of the limitations is largely missing:

- Under what conditions yields the PCA the dimensionality of a data set? (When can we assume the data follows a multivariate Gaussian distribution?). Is that the case for neural data? Why are your findings relevant for neuroscience?

- What are the limitations of a linear response theory? When would it break down?

- What is the relationship between ‘linear rate units’ and ‘neurons’. (Often ‘rate units’ are considered as populations of neurons. But how does this fit together with the reference to single neuron motifs (e.g. Song et al. 2005?))

- The relevance of the investigation of g approaching 1 from below is not clear. Isn’t at g=1 the network dynamics turning unstable and the linear response picture breaking down? Already close to g=1, the fluctuations are getting large, so one might expect that the linear response description is getting increasingly inaccurate because \\phi(x)≠x. So while the power-law scaling is an interesting observation, I am not sure it has any relevance for neuroscience (but of course, I’d love to be proven wrong on this).

* While ‘the equations speak for themselves’, an intuitive explanation of the observed results and the underlying mechanism is largely missing:

- Why does g->1 lead to a few dominant eigenvalues in the covariance matrix?

- Why do antisymmetry networks have a qualitatively different shape of the distribution of eigenvalues?

- Why do (anti)symmetry networks results in lower (higher) relative dimension D/N? Why does dependence of D(\\kappa) change when fixing g_r?

-

The Discussion section seems to add more new results (e.g. reference to ring network and other results in the Supplementary Material) instead of discussing the interpretation of the results and implications for theory and experiments.

Minor Comments:

* Consider changing the title to “The spectrum of covariance matrices of neuronal networks with linear dynamics” or “The covariance spectrum of recurrent neural networks with linear dynamics” or another title that contains the word 'linear' or 'linearly' would make it more clear that here a linear response framework is being used. This also leaves space for future studies that explore nonlinear regimes.

* Figure labels are so small that they are too small. Please increase the label and legend font size to make it readable.

* Make clear that PCA is based on pairwise statistics, so it doesn’t require simultaneous recording of all N neurons, it would be enough (assuming stationarity) for all pairs to be recorded one after the other. Therefore, I would like to politely disagree with the notion of 'local' and 'global' features, they might mislead some readers as only two-point interactions are considered here.

* The comparison of the experimentally obtained covariance spectra of zebrafish data to analytically obtained ones includes assumptions about the temporal correlations of the data (Marchenko-Pastur assumes that samples are independent, i.e. sampled at time frames that are sufficiently far apart such that temporal correlations can be ignored, to what extend is that assumption justified in your data? (It would be helpful to report the frame rate in the caption of the figure 8 and the decay time constant of GCamp6f and the number of time frames used to calculate the empirical covariance matrix. I think your reference 10 has a frame rate of 2.11 per s and GCamp6f has a decay constant of 1796±73ms according to (https://www.ncbi.nlm.nih.gov/pmc/articles/PMC3777791/#SD1) but please check.

* The experimentally recorded zebrafish was exposed to visual stimuli and the data was z-scored according to your methods, please comment on how both spatiotemporally structured external input and z-scoring affects your results.

* Line 126 should read semi-positive instead of positive.

* Line 139: Cite earlier papers on participation ratio.

* Line 160: “Matches pretty well” use precise language.

* Line 167/Figure 1A,B: showing more than one network realization would be more convincing.

* Figure 1: labels and legend too small

* Figure 1A,B: consider doing a stair step graph for the empirical histogram across multiple (e.g. 10) network realizations

* Line 187: c_0 seems not to be introduced in the main text.

* When you refer to Supplement, it would help if you refer to the section of the supplement because it is 60 pages long so things would be easier to find.

* Figure 5: inset too small, axes not legible

* Line 296: only K<<n assumed="" explicitly="" is="">>1 for the Gaussian assumption.

* It would be nice to plot both D(f) and D(\\alpha) (equation 23) next to figure 7AB so the effect of sparse time sampling becomes even visually more obvious.

* A closer look at the methods section (5.6) indicates that the authors incorporated temporal sampling corrections in their calculation of the theoretical spectral CDFs used to fit the neural data. However, it is not clear in the main text (3.8) that they did this.

* In section 3.8, where the authors fit distributions to data, a discussion of the data is missing. Would you expect the same PCA distribution for spikes instead of calcium? What is the effect of the Calcium response? It would be good to comment on the fact that the eigenvalues of a covariance matrix change after a change of variable.

* In figure 8, important details are missing: How many data points are used to estimate the covariance matrix? Why don’t you fit the model including symmetry/antisymmetry? What D/N do you numerically estimate for the different clusters? Do you have full access to all neurons Calcium activity? If not, do you apply your spatial sparsity model?

* Instead of motifs-> second-order motifs. (there are also higher order motifs)

* Please go carefully over all references to correct typos and misspelled names.

Stylistic:

* “iid” -> “i.i.d.”

* Highly complex->complex

* “Frequency spectrum in Fourier transform”-> “Frequency spectrum obtained via Fourier transform”

* “co-fluctuations” doesn’t to my knowledge exist, maybe use covariation instead?

* Instead of “the network’s Principal Component Analysis, we would propose the network dynamics Principal Component Analysis.

* Line 237: For denoting the imaginary i, I would not use a double-struck i.

Typos:

* Whitespace line 71 after dynamics.

* Excitatory-Inhibitory-> excitatory-inhibitory

* Line 559: “random matrix” -> “random matrices”

* In abstract: “The theoretical results are compared with those from finite-size networks and and the effects” should read “The theoretical results are compared with those from finite-size networks and the effects”.

* “the rank plot with exponent”

* “supported by a NIH grant” -> “supported by an NIH”

* “which correspond to overabundance of certain subgraphs” -> “which correspond to an overabundance of certain subgraphs”

* “as g approach the critical”-> “as g approaches the critical”

* “In large-network limit”-> “In the large-network limit”

* “the error can also be measure under” -> “the error can also be measured under”

* “Storing Infinite Numbers of Patterns in a Spin-Galss Model of Neural Networks”-> “Storing Infinite Numbers of Patterns in a Spin-Glass Model of Neural Networks”

* “Multiplciation of certain noncommuting random variables”. -> “Multiplication of certain noncommuting random variables”.

Rainer Engelken</n>

Reviewer #2: Summary:

--------

In this manuscript, Hu and Sompolinsky derive analytical expressions

for the eigenvalue spectrum of covariance matrices and the related

dimensionality measure (participation ratio) for random networks (iid

weights / motifs / excitatory-inhibitory, and also some structured

networks for comparison). Their theory is based on linear(ized)

dynamics, where there is a simple relation between covariances and

connections. Yet, for the general case of non-normal connectivity

matrices, the derivation of the covariance spectrum from connectivity

parameters is involved. Their theoretical derivations, that for better

readability are mostly presented in the extensive supplement, are

based on the seminal work by Sommers, Crisanti, Sompolinsky and Stein

in 1988.

Given the large interest in dimensionality of neural activity in the

computational neuroscience community over the recent years, this work

is highly relevant and timely and an important contribution to the

understanding of the relation between structure and dynamics in neural

networks. The presentation is mostly clear, the theoretical

derivations are solid and the authors also show an application to

experimental data (whole-brain calcium imaging data from larval

zebrafish). We therefore believe this paper should eventually be

published in PLOS CB. There are, however, a few major and minor issues

(see below) that need to be resolved in a revised version before the

manuscript is ready for publication.

Major points:

-------------

-------------

Effect of other motifs:

-----------------------

The authors study in depth the effect of reciprocal connections /

symmetry of connections on the covariance spectrum. They also discuss

the other motifs (divergent, convergent, chain), but state that they

do not "affect the bulk spectrum of C" (main text line 220). In the

supplement below Eq. S26 the authors, however, state that also

divergent, convergent and chain motifs affect the bulk spectrum

indirectly by channeling their effect through changing the normalized

reciprocal motifs. Can the authors clarify this and elaborate in more

detail in the main text, which motifs affect the bulk spectrum in

which way? Also the critical coupling strength / stability should

depend on all motifs (see Eq. S26), so one would expect an influence

of all motifs on the bulk covariance spectrum.

Clarification of limitations:

-----------------------------

In Section 3.5 the authors should stress more that their theoretical

predictions are limited to networks where all connections have identical

variance. Their choice of E-I network is a special case that is

explicitly constructed such that this requirement holds. In typical

E-I networks the variance is not the same due to different population

sizes, connection probabilities, and synaptic strengths (that are not

precisely tuned to achieve the same variance of E and I connections).

Relation to Sommers et al. 1988 (S1988):

----------------------------------------

In supplement S1 the authors show how to derive the probability

density p_c for the covariance eigenvalues from the potential Phi that

has been computed in S1988. What did not become clear to us is how the

derivation deviates from the calculations in S1988. Because S1988

calculated the probability density p_J of the connectivity spectrum

rather than p_c. Both calculations seem to rely on the precision

matrix, but the detailed differences in calculations did not become

clear from reading the supplement. Can the authors please provide more

details in which steps of the calculations results from S1988 are

exactly used and in which steps results needed to be adapted to treat

the covariance spectrum rather than the connectivity spectrum?

Relation to Stringer, Pachitariu et al. Nature 2019 (SP2019)

------------------------------------------------------------

In line 188, the authors present their results as an alternative

mechanism for the experimentally observed power laws of covariance

spectra in SP2019. The authors, however, study the covariance

structure of spontaneous network dynamics while SP2019 studies

stimulus-evoked activity (responses in V1 due to visual stimuli).

Would one thus not expect differences in covariance spectra? Can the

authors briefly elaborate on this?

Relation to Dahmen, Recanatesi et al. biorxiv 2020 (DR2020):

------------------------------------------------------------

In line 208 the authors state that the dimensionality result Eq. 10

can also be derived based on results of their reference [11]. This has

already been done in DR2020

(https://www.biorxiv.org/content/10.1101/2020.11.02.365072v1.full.pdf).

It would be helpful to explain the relation of the current work to

these parallel developments. This seems particularly important given

the high similarities between figures of the current manuscript and

figures in DR2020, in particular

-Fig 1D <-> DR2020:Fig 2B

-Fig 4B <-> DR2020:Fig 4B

The approximation in l.257 for D/N as a function of g_r corresponds to

the approximate result in DR2020.

Also in the supplement "First two moments as a corollary of results in

[3]" it would be helpful of the authors cited DR2020 to clarify

the close relation of these parallel works.

Motivation of the manuscript:

------------------------------------------------

To motivate their work, the authors dinstinguish in the first

paragraph of the introduction between local and global features of

dynamics. They cite a few works falling in the local feature category

and argue that their work targets the global scale.

We, however, disagree with the authors' categorization.

While their refs [55,48] really discuss local features,

refs [27,11,18] study correlations (mean and variance across populations).

The latter emerge from collective network effects and should thereby be

attributed to the global feature category. In fact, since the dimensionality

and covariance spectrum are based on the very same covariances, the

authors' work falls in the same category as [27,11,18].

Minor points:

-------------

-------------

Power law exponent:

-------------------

Why is the power law shape of the covariance spectrum not continuous

in the parameter kappa? I.e. why is there a single power law exponent

for -1<kappa<1, a="" all="" and="" at="" but="" different="" for="" kappa="1" none="" one="">kappa=-1? Are these results an artefact of the calculations that are

different for kappa \\in {-1,1} and -1<kappa<1? authors="" did="" perform="" the="">numerical comparisons for the power law exponents for various kappa?

The authors should extend their discussion on these points.

Wrong cross-references in the supplement:

-----------------------------------------

Please check again the cross-references in the supplement. Some of

them textually refer to Figures in the main text, but the hyperlink

takes us to figures in the supplement, e.g. in l.474: Ref to Fig 6

(in the main text) wrongly links to Fig. S6 in the supplement.

Figures:

--------

Fig 1: Colors for theory and simulation not consistent across panels

Fig 4A, Fig 5C&D, Fig 6A&B: legend labels for lines not consistent:

decide for "theory" or "Gaussian theory"

Fig. 6: Better explain the correction with the modified connection weight.

Fig. S6: y-axis labels are cut off

Strong non-normal effects:

--------------------------

Sections S5.2 and S5.3: I am not sure which point the authors want to

make here. The bimodality of the spectrum is an interesting

observation, but the comparison to the covariance spectrum of the

matching normal connectivity shows that the bimodality is not an

effect of the non-normality. In contrast the bimodality is more severe

for the matched normal. So the non-normality seems to suppress the

bimodality. If this is the main message then the authors should write

this more clearly in the supplement and also in Sec 3.3.2 of the main

text. How do the covariance spectra and matched normal spectra compare

for kappa_re>0?

Time and space sampling:

------------------------

We found supplemental sections S8 and S9 hard to follow, especially

for readers that are not familiar with free probability theory. We are

aware that the authors cannot go into all details on this issue in

this manuscript, but it would be helpful for the average reader of

PLOS CB if the authors could provide even more intuition on the

various steps of the calculations and possibly single out a good

references for an overview article that introduces the concept

of free prbability theory.

References:

-----------

l.272: We think it would be suitable to also cite Schuessler et al.

NeurIPS 2020, which showed that training random networks creates

low-rank connectivity components.

More detailed minor points (Supplement):

----------------------------------------

----------------------------------------

Eq.S3: indicate that -z is the argument corresponding to epsilon in

Eq.S2, i.e. d Phi/d epsilon(-z,\\eta)

In Eq. S2 epsilon shall be larger than zero in, but z can take on all

values in C excluding the real line. Could you please explain this a

bit more in detail?

l.165-166: Please define F_C also here in supplement.

Eq.S26: It it worth noting that \\hat{kappa}_*(J) is normalized by the

variance of J and \\hat{kappa}_*(\\tilde{J}) is normalized by the

variance of \\tilde{J}

Eq.S27: It is not clear where Sylvester's identity has been applied.

What is A and B in eq. S27?

l.340-341: z=eta? Please remind the reader here on the relation

between p_C and p_eta (shown in l.81).

Eq.S66: How to get from mu_1 to mu_1(C)?

l.426 and 436: Ref to Fig 3B correct?

Eq.S75: dx missing on the rhs?

l.513-514: what are overlines over omega and C referring to?

Typos (Main text):

------------------

l.22: and and -> and

l.125: the -> The

l.140: two moments -> two moments of

l.272: .[33,44]. -> [33,44].

l.528: broken sentence

l.536: weights) -> weights

l.559: random matrix -> random matrix theory

l.619: pdf -> cdf

l.623: measure -> measured

Typos (Supplement):

-------------------

l.121: E(\\lambda)^n -> E(\\lambda^n)

l.129: the its -> its

l.208: comma missing in between inline equations

l.354 and in following sections: p_P(x)->p_eta(x)

l.476: w0 ->w_0

l.523: C\\omega)->C(\\omega)

l.533-534: g missing in equation?</kappa<1?></kappa<1,>

Reviewer #3: The manuscript analyzed theoretically the eigenvalue distribution of covariance matrix of the spontaneous neural activities in the randomly connected recurrent neuronal networks, and found the distribution has a finitely supported smooth bulk spectrum and exhibits an approximated power-law tail for coupling matrices near the critical edge. The simulation results consist with the theoretical prediction. I believe the mathematical derivations are rigorous and the results are solid.

However, I have some major concerns.

1. To analyze the eigenvalue distribution of covariance matrix, the authors made some important simplifications, for example, both the interactions between neurons and local dynamics of single neurons are linear. However, the local dynamics and interactions of our neural systems are all nonlinear, and lots of computing advantages are emerged based on these nonlinear characteristics. So could the authors provide some more simulation results to support that the main results can be extended to the nonlinear neural networks.

2. I am not clear what the eigenvalue distributions could give some new insights to the neural information processing mechanism? For example, the first few eigenvalues and eigenvectors of the covariance matrix can reflect the main computing characteristic or features of neural activities, so could the authors address the new computing advantages or functional meaning reflected by the eigenvalue distributions to the dynamics of neural activities?

3. The manuscript analyzed the covariance matrix of spontaneous activity, however, the neural systems usually show persistent activities when our brain performs some cognitive functions. Could the results extend to the study of persistent activities?

**********

**Have the authors made all data and (if applicable) computational code underlying the findings in their manuscript fully available?**

The PLOS Data policy requires authors to make all data and code underlying the findings described in their manuscript fully available without restriction, with rare exception (please refer to the Data Availability Statement in the manuscript PDF file). The data and code should be provided as part of the manuscript or its supporting information, or deposited to a public repository. For example, in addition to summary statistics, the data points behind means, medians and variance measures should be available. If there are restrictions on publicly sharing data or code —e.g. participant privacy or use of data from a third party—those must be specified.

Reviewer #1: Yes

Reviewer #2: Yes

Reviewer #3: Yes

**********

PLOS authors have the option to publish the peer review history of their article (what does this mean?). If published, this will include your full peer review and any attached files.

If you choose “no”, your identity will remain anonymous but your review may still be made public.

**Do you want your identity to be public for this peer review?** For information about this choice, including consent withdrawal, please see our Privacy Policy.

Reviewer #1: **Yes: **Rainer Engelken

Reviewer #2: No

Reviewer #3: No

Figure Files:

While revising your submission, please upload your figure files to the Preflight Analysis and Conversion Engine (PACE) digital diagnostic tool, https://pacev2.apexcovantage.com. PACE helps ensure that figures meet PLOS requirements. To use PACE, you must first register as a user. Then, login and navigate to the UPLOAD tab, where you will find detailed instructions on how to use the tool. If you encounter any issues or have any questions when using PACE, please email us at figures@plos.org.

Data Requirements:

Please note that, as a condition of publication, PLOS' data policy requires that you make available all data used to draw the conclusions outlined in your manuscript. Data must be deposited in an appropriate repository, included within the body of the manuscript, or uploaded as supporting information. This includes all numerical values that were used to generate graphs, histograms etc.. For an example in PLOS Biology see here: http://www.plosbiology.org/article/info%3Adoi%2F10.1371%2Fjournal.pbio.1001908#s5.

Reproducibility:

To enhance the reproducibility of your results, we recommend that you deposit your laboratory protocols in protocols.io, where a protocol can be assigned its own identifier (DOI) such that it can be cited independently in the future. Additionally, PLOS ONE offers an option to publish peer-reviewed clinical study protocols. Read more information on sharing protocols at https://plos.org/protocols?utm_medium=editorial-email&utm_source=authorletters&utm_campaign=protocols

# Author comment: Author response to Decision Letter 0

**DOI:** 10.1371/journal.pcbi.1010327.r002

19 Apr 2022

Attachment Submitted filename: response to reviewers comments.pdf Click here for additional data file.

# Peer review: Decision Letter 1

**DOI:** 10.1371/journal.pcbi.1010327.r003

**Authors:** Thomas Serre, Tianming Yang

1 Jun 2022

Dear Dr. Hu,

Thank you very much for submitting your manuscript "The spectrum of covariance matrices of randomly connected recurrent neuronal networks with linear dynamics" for consideration at PLOS Computational Biology. As with all papers reviewed by the journal, your manuscript was reviewed by members of the editorial board and by several independent reviewers. The reviewers appreciated the attention to an important topic. Based on the reviews, we are likely to accept this manuscript for publication, providing that you modify the manuscript according to the review recommendations.

Please prepare and submit your revised manuscript within 30 days. If you anticipate any delay, please let us know the expected resubmission date by replying to this email.

When you are ready to resubmit, please upload the following:

[1] A letter containing a detailed list of your responses to all review comments, and a description of the changes you have made in the manuscript. Please note while forming your response, if your article is accepted, you may have the opportunity to make the peer review history publicly available. The record will include editor decision letters (with reviews) and your responses to reviewer comments. If eligible, we will contact you to opt in or out

[2] Two versions of the revised manuscript: one with either highlights or tracked changes denoting where the text has been changed; the other a clean version (uploaded as the manuscript file).

Important additional instructions are given below your reviewer comments.

Thank you again for your submission to our journal. We hope that our editorial process has been constructive so far, and we welcome your feedback at any time. Please don't hesitate to contact us if you have any questions or comments.

Sincerely,

Tianming Yang

Associate Editor

PLOS Computational Biology

Thomas Serre

Deputy Editor

PLOS Computational Biology

***********************

A link appears below if there are any accompanying review attachments. If you believe any reviews to be missing, please contact ploscompbiol@plos.org immediately:

[LINK]

Reviewer's Responses to Questions

**Comments to the Authors:**

**Please note here if the review is uploaded as an attachment.**

Reviewer #1: General evaluation:

I thank the authors for addressing all major comments and questions. The manuscript is now much more clear and understandable. The additional figures greatly improve the presentation. I agree with the change of the title and consider the finding sufficient for publication in this journal.

There are just one minor issue remaining:

Minor comment:

Line 264: Instead of "Since J here is a normal matrix, this qualitative difference from the i.i.d. random connectivity can be understood considering the eigenvalues of J [49], which all lie on the imaginary axis and never approach 1 to cause large eigenvalues in the covariance when increasing g."

I would suggest writing instead for enhanced clarity: "Since J here is a normal matrix, this qualitative difference from the i.i.d. random connectivity can be understood considering the eigenvalues of J[49]. The eigenvalues of J all lie on the imaginary axis and therefore never approach 1. Thus, the eigenvalues of the corresponding covariance matrix don't develop a long tail."

Just for clarification: Our previous comment about line 296 was erroneous.

Reviewer #2: We thank the authors for addressing most of our comments in detail in

the revision. The presentation of results has much improved. There

are, however, a few points that are still unclear and need to be

clarified before the manuscript is ready for publication in PLOS CB.

Power law approximation:

------------------------

We apologize that parts of our previous question on the power-law

approximation seem to have been cut during the submission of the

comments. The missing part concerned the non-continuousness of the

power law exponent with kappa. We find unintuitive that there seems to

be an abrupt change in the distribution of bulk eigenvalues from e.g.

kappa=1- to kappa=1 (likewise for the other extreme case kappa=-1).

Because the approximated theory predicts different power law

exponents. Can the authors show simulations of p_c in logarithmic

yscale for readers to see what happens to the power law for fixed g_r

and varying kappa over the whole range including the edge cases

kappa=-1 and kappa=1 (Similar to Fig 4D but with log scale on y axis

and including cases closer and including kappa=+-1)? We understand

that the topology of connectivity eigenvalues changes from 2D to 1D at

the limiting kappas, but this happens in a smooth manner, so we would

have thought a smooth change in the covariance spectra.

Nonlinear network:

------------------

The analysis of the nonlinear network is a nice and convincing

addition to the manuscript. We, however, do find the authors'

description in lines 514ff potentially misleading. There the authors

state:

"Note that even for g > 1, the dynamics examined here is largely

driven by the noise rather than the intrinsic chaotic dynamics studied

in [50]."

It is not surprising that the linear approximation of the dynamics and

spectrum work well even for the case g>1 studied in Fig. 9. The

dynamics of all networks shown in Fig. 9 are not chaotic. In fact, the

dynamics are even linearly stable. As shown in Molgedey et al. PRL

1992 for discrete-time networks and in Schuecker et al. PRX 2018 for

continuous dynamics (same model as studied in this manuscript) the

transition to linear instability and the transition to chaos are both

shifted to larger values of g in the presence of external input (cf.

e.g. Fig. 3 in Schuecker et al.). Maybe the authors had this in mind,

but they should make the statement more precise to not suggest that

the approximation works well for networks with chaotic dynamics.

Minor points:

-------------

The xlabel in Fig 1D is missing.

Typos:

------

l479: a concise theoretical characterizations -> concise theoretical characterizations

l619: firing rates r_i(t) is binned -> firing rates r_i(t) are binned

Supp l.215f: This does not contradicting -> This does not contradict

Supp l.217: one introduce -> one introduces

Reviewer #3: I thank the authors for a thorough revision and comprehensively answering all of my questions. I have no further concerns.

**********

**Have the authors made all data and (if applicable) computational code underlying the findings in their manuscript fully available?**

The PLOS Data policy requires authors to make all data and code underlying the findings described in their manuscript fully available without restriction, with rare exception (please refer to the Data Availability Statement in the manuscript PDF file). The data and code should be provided as part of the manuscript or its supporting information, or deposited to a public repository. For example, in addition to summary statistics, the data points behind means, medians and variance measures should be available. If there are restrictions on publicly sharing data or code —e.g. participant privacy or use of data from a third party—those must be specified.

Reviewer #1: Yes

Reviewer #2: Yes

Reviewer #3: Yes

**********

PLOS authors have the option to publish the peer review history of their article (what does this mean?). If published, this will include your full peer review and any attached files.

If you choose “no”, your identity will remain anonymous but your review may still be made public.

**Do you want your identity to be public for this peer review?** For information about this choice, including consent withdrawal, please see our Privacy Policy.

Reviewer #1: **Yes: **Rainer Engelken

Reviewer #2: No

Reviewer #3: **Yes: **Yuanyuan Mi

Figure Files:

While revising your submission, please upload your figure files to the Preflight Analysis and Conversion Engine (PACE) digital diagnostic tool, https://pacev2.apexcovantage.com. PACE helps ensure that figures meet PLOS requirements. To use PACE, you must first register as a user. Then, login and navigate to the UPLOAD tab, where you will find detailed instructions on how to use the tool. If you encounter any issues or have any questions when using PACE, please email us at figures@plos.org.

Data Requirements:

Please note that, as a condition of publication, PLOS' data policy requires that you make available all data used to draw the conclusions outlined in your manuscript. Data must be deposited in an appropriate repository, included within the body of the manuscript, or uploaded as supporting information. This includes all numerical values that were used to generate graphs, histograms etc.. For an example in PLOS Biology see here: http://www.plosbiology.org/article/info%3Adoi%2F10.1371%2Fjournal.pbio.1001908#s5.

Reproducibility:

To enhance the reproducibility of your results, we recommend that you deposit your laboratory protocols in protocols.io, where a protocol can be assigned its own identifier (DOI) such that it can be cited independently in the future. Additionally, PLOS ONE offers an option to publish peer-reviewed clinical study protocols. Read more information on sharing protocols at https://plos.org/protocols?utm_medium=editorial-email&utm_source=authorletters&utm_campaign=protocols

References:

Review your reference list to ensure that it is complete and correct. If you have cited papers that have been retracted, please include the rationale for doing so in the manuscript text, or remove these references and replace them with relevant current references. Any changes to the reference list should be mentioned in the rebuttal letter that accompanies your revised manuscript.

*If you need to cite a retracted article, indicate the article’s retracted status in the References list and also include a citation and full reference for the retraction notice.*

# Author comment: Author response to Decision Letter 1

**DOI:** 10.1371/journal.pcbi.1010327.r004

14 Jun 2022

Attachment Submitted filename: response to reviewers comments_2.pdf Click here for additional data file.

# Editor report: Decision Letter 2

**DOI:** 10.1371/journal.pcbi.1010327.r005

**Authors:** Thomas Serre, Tianming Yang

24 Jun 2022

Dear Dr. Hu,

We are pleased to inform you that your manuscript 'The spectrum of covariance matrices of randomly connected recurrent neuronal networks with linear dynamics' has been provisionally accepted for publication in PLOS Computational Biology.

Before your manuscript can be formally accepted you will need to complete some formatting changes, which you will receive in a follow up email. A member of our team will be in touch with a set of requests.

Please note that your manuscript will not be scheduled for publication until you have made the required changes, so a swift response is appreciated.

IMPORTANT: The editorial review process is now complete. PLOS will only permit corrections to spelling, formatting or significant scientific errors from this point onwards. Requests for major changes, or any which affect the scientific understanding of your work, will cause delays to the publication date of your manuscript.

Should you, your institution's press office or the journal office choose to press release your paper, you will automatically be opted out of early publication. We ask that you notify us now if you or your institution is planning to press release the article. All press must be co-ordinated with PLOS.

Thank you again for supporting Open Access publishing; we are looking forward to publishing your work in PLOS Computational Biology. 

Best regards,

Tianming Yang

Associate Editor

PLOS Computational Biology

Thomas Serre

Deputy Editor

PLOS Computational Biology

***********************************************************

# Editor report: Acceptance letter

**DOI:** 10.1371/journal.pcbi.1010327.r006

**Authors:** Thomas Serre, Tianming Yang

15 Jul 2022

PCOMPBIOL-D-21-02214R2 

The spectrum of covariance matrices of randomly connected recurrent neuronal networks with linear dynamics

Dear Dr Hu,

I am pleased to inform you that your manuscript has been formally accepted for publication in PLOS Computational Biology. Your manuscript is now with our production department and you will be notified of the publication date in due course.

The corresponding author will soon be receiving a typeset proof for review, to ensure errors have not been introduced during production. Please review the PDF proof of your manuscript carefully, as this is the last chance to correct any errors. Please note that major changes, or those which affect the scientific understanding of the work, will likely cause delays to the publication date of your manuscript. 

Soon after your final files are uploaded, unless you have opted out, the early version of your manuscript will be published online. The date of the early version will be your article's publication date. The final article will be published to the same URL, and all versions of the paper will be accessible to readers.

Thank you again for supporting PLOS Computational Biology and open-access publishing. We are looking forward to publishing your work! 

With kind regards,

Zsofia Freund

PLOS Computational Biology | Carlyle House, Carlyle Road, Cambridge CB4 3DN | United Kingdom ploscompbiol@plos.org | Phone +44 (0) 1223-442824 | ploscompbiol.org | @PLOSCompBiol
